# The Contrasting Role of Fire in Shaping Landscape Genetic Patterns of Small Mammals Across Two Islands

**DOI:** 10.1002/ece3.73320

**Published:** 2026-03-26

**Authors:** Alexander R. Carey, Teigan Cremona, Georgina Neave, Hugh F. Davies, Brett P. Murphy, Geoffrey J. Cary, Tiwi Rangers, Sam C. Banks

**Affiliations:** ^1^ Research Institute for the Environment and Livelihoods, Faculty of Science and Technology Charles Darwin University Casuarina Northwest Territories Australia; ^2^ School of Environmental and Rural Science University of New England Armidale New South Wales Australia; ^3^ Fenner School of Environment & Society The Australian National University Canberra Australian Capital Territory Australia; ^4^ Tiwi Resources Pty Ltd Casuarina Northwest Territories Australia

**Keywords:** connectivity, conservation genetics, disturbance ecology, fire, landscape genetics

## Abstract

Population connectivity maintains genetic diversity and underpins adaptive capacity and long‐term persistence. When assessing connectivity, landscape genetic analyses have largely focused on static features such as topography and have rarely incorporated disturbance regimes like fire, which may also shape genetic connectivity. We assessed genetic diversity and structure for three declining mammal species in northern Australian savannas: the northern brown bandicoot ( *Isoodon macrourus* ), northern brushtail possum (*Trichosurus vulpecula arnhemensis*) and black‐footed tree‐rat (*Mesembriomys gouldii melvillensis*). Using genetic distance data, we optimised resistance surfaces to evaluate how landscape variables, including fire history, rainfall, vegetation, topography, watercourses, and feral species, influence gene flow. Analyses were conducted at fine (< 20 km between individuals) and broad (whole‐island) scales across Bathurst (up to 60 km) and Melville (up to 120 km) Islands, which differ markedly in disturbance regimes. Genetic patterns and their drivers varied by species, island and scale. The influence of fire was most apparent on Bathurst Island, where feral predators and herbivores are less abundant and rainfall is high. Northern brown bandicoots showed weak broad‐scale genetic structure on Bathurst Island, while high fire frequency reduced connectivity at fine scales. For northern brushtail possums, high rainfall reduced genetic connectivity at both broad and fine scales. On Melville Island, where multiple interacting threats of fire, feral predators and herbivores are more prevalent, topographic ruggedness promoted connectivity for northern brown bandicoots and northern brushtail possums at broad scales. At fine scales, geographic distance best explained the genetic patterns for these species. For black‐footed tree‐rats, which occur only on Melville Island, broad‐scale connectivity was reduced in low fire frequency areas (e.g., plantation and mangrove), while at fine scales low rainfall and high ruggedness were associated with reduced gene flow. Our results show that fire can influence resistance to gene flow, but effects are species‐ and context‐dependent. Effective conservation requires accounting for species‐specific ecology and local disturbance regimes when evaluating connectivity. Fire management should be a priority where fire strongly structures gene flow, while landscapes with multiple interacting threats require broader strategies including feral species control and habitat protection. Our study underscores the value of incorporating disturbance regimes into landscape genetics to guide context‐specific conservation.

## Graphical abstract

We evaluated how landscape features, including fire, influence genetic connectivity for three small mammal species in northern Australian savannas. Using resistance surface modelling across two islands with contrasting disturbance regimes, we found that fire, rainfall, and topography affected gene flow in species‐ and scale‐specific ways. Our results highlight the importance of incorporating disturbance regimes into landscape genetic analyses to inform targeted conservation strategies.

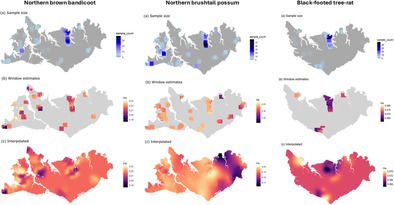

## 1 Introduction

The maintenance of genetic diversity and connectivity between populations is important for species persistence (Taft et al. 2020). Genetic information can therefore greatly improve the management of threatened species. Genetic patterns, including the spatial distribution of genetic diversity and differentiation between populations across a landscape, are shaped by a complex interaction between a species' ecology, such as dispersal, life history, and habitat preferences, and the surrounding landscape, including habitat configuration, disturbance regimes, and interspecies interactions (Paz et al. 2015). An understanding of the drivers of genetic patterns is fundamental for making informed management decisions to conserve genetic diversity.

Landscape genetic analyses seek to identify the variables that facilitate or impede movement between populations (Manel et al. 2003; Manel and Holderegger 2013). In identifying the variables driving genetic patterns, it is possible to provide managers with information about refugia, dispersal pathways, and areas with increased inbreeding and genetic drift (Shaw et al. 2023). Most commonly, landscape genetic studies investigate population connectivity across static landscape elements, as genetic patterns require a certain amount of time to reach a perceivable equilibrium (Epps and Keyghobadi 2015). However, disturbances and habitat succession have been demonstrated to influence spatial genetic patterns and are therefore important to include in landscape genetic analyses (Banks et al. 2013).

Fire is often a major driver of genetic patterns due to the influence of habitat dynamics on demographic processes (Banks et al. 2012, 2013). By shaping habitat structure, resource availability, and community composition, fire influences dispersal and reproduction and, therefore, the distribution of genetic diversity within and among populations (Banks et al. 2012, 2013). This is a continual process following the habitat dynamics of post‐fire environments. Human‐driven changes to climate and land use have altered global fire regimes, which are expected to intensify further, with increases in frequency, severity and size (Sayedi et al. 2024). These altered fire regimes, in particular increased fire frequency, have been identified as a threatening process for many species (Kelly et al. 2020). Understanding the ecological and genetic consequences of fire is therefore essential. Research into the genetic consequences of fire in animal populations has been limited but is growing, providing crucial information for species conservation and landscape management (Harris et al. 2023; Sitters and Di Stefano 2020).

Savannas, the world's most frequently burnt biome, cover over 1.9 million km^2^ of northern Australia, supporting species generally well adapted to fire (Andersen et al. 2012; Beringer et al. 2015). However, widespread declines in small mammal (< 5 kg) populations since the mid to late 20th century have been linked to the disruption of traditional Indigenous fire management, and the impacts of feral predators and herbivores (Stobo‐Wilson et al. 2020a, 2020b). Feral cat predation is now considered the primary driver of these declines in northern Australia, with contemporary fire regimes and feral herbivore grazing simplifying habitats, thereby increasing hunting efficiency and, in some cases, cat abundance (Hradsky 2020; Leahy et al. 2016; Griffiths et al. 2015). Applying landscape genetic analyses in fire‐prone systems offers a valuable means of identifying drivers of genetic structure to inform effective management strategies.

The principal aim of this study is to determine the role of fire in shaping genetic connectivity. More specifically, we aim to understand how the effect of fire on genetic connectivity may differ between species, with disturbance severity and scales of observation. We collated genetic data collected from across the Tiwi Islands, Bathurst and Melville, in Northern Territory, Australia, to conduct an island‐wide assessment of the genetic patterns of three native mammal species and identify drivers of landscape genetic structure at fine and broad spatial scales. Bathurst and Melville Island exhibit contrasting disturbance regimes, with Bathurst Island broadly characterised as being less degraded than Melville Island due to minimal habitat loss from land clearing, lower feral cat density, and the absence of large feral herbivores. These islands therefore offer a unique natural contrast with which to compare the genetic patterns of small mammals highly susceptible to such disturbances. Using multiple species with different habitat requirements, life histories, and dispersal capacities further allowed us to understand the differing management requirements. We asked three key questions: (1) How do spatial patterns of genetic diversity differ between species and islands? (2) How do spatial patterns of genetic structure differ between species and islands? (3) What landscape variables, such as fire history, rainfall, vegetation, topography, watercourses or feral species, are driving genetic patterns and do they differ between species, islands and at fine and broad spatial scales?

We hypothesised that genetic patterns and their key drivers differ between species due to associations between species ecology and landscape variables. For example, more dispersive and habitat generalist species (e.g., northern brown bandicoots, *Isoodon macrourus* ) will show reduced landscape structure compared with species with reduced dispersal distance (e.g., northern brushtail possums, *Trichosurus vulpecula arnhemensis*) or specialist habitat requirements (e.g., black‐footed tree‐rats, *Mesembriomys gouldii melvillensis*). We hypothesised that the island with heightened threats, Melville Island, would show greater genetic structure patterns driven by threats such as frequent fire, and feral cat and herbivore activity. We hypothesised that disturbance regimes (i.e., fire) would play an important role in shaping genetic patterns with areas of high fire frequency, or of frequent late dry season fires, reducing genetic connectivity. Lastly, we hypothesised that the broad scale analysis would identify more stable landscape features, such as topography, as longer‐term drivers of genetic connectivity whilst the fine scale analysis would identify more dynamic disturbances within savanna habitat, such as fire and feral species, as more recent drivers of genetic connectivity.

## 2 Methods

### 2.1 Study Site

**FIGURE 1:**
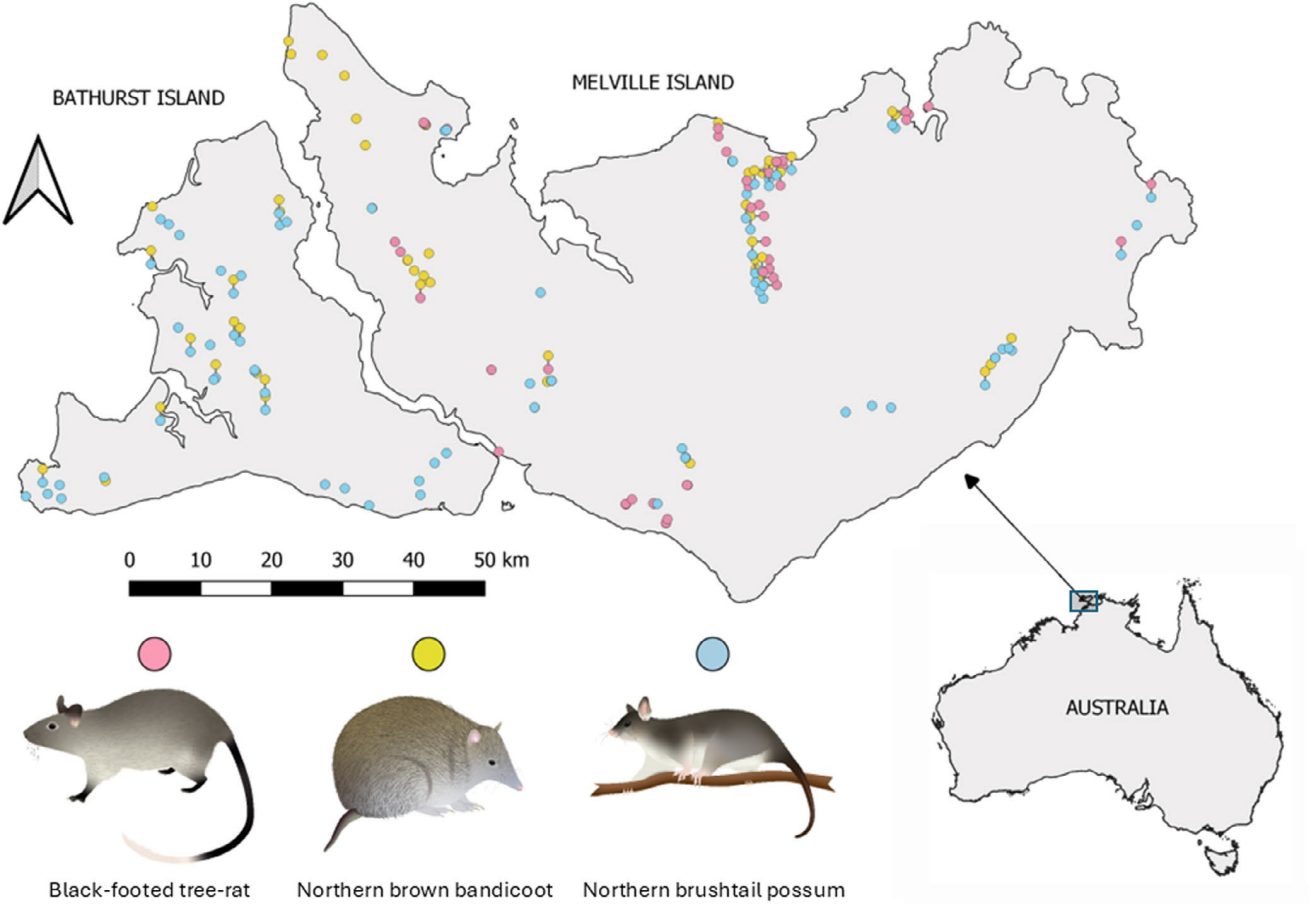
Genetic samples of three small mammal species collected from the Tiwi Islands between 2018 and 2023.

The Tiwi Islands are located approximately 50 km north of Darwin, in Australia's Northern Territory within the tropical savanna biome and consist of two main islands: Bathurst (1639 km^2^) and Melville (5786 km^2^) Islands (Figure 1). As a result of rapid sea level rise between 12,000 and 8000 years ago, the islands were split from the mainland and each other (Firth et al. 2006). Isolation from the mainland has contributed to the Tiwi Islands remaining as important refuges for many small mammal species. Only the very narrow Apsley Strait (typically < 500 m) separates Bathurst and Melville Islands, and they therefore have a similar history and environments (Firth et al. 2006). Both islands are of low elevation (< 150 m above sea level) and are topographically simple. However, important distinctions exist that allow an interesting comparison of the landscape features and ecological context that shape the genetic patterns of small mammals.

Melville Island has recently documented declines in small mammal populations, likely driven by the same threatening processes implicated in the declines on mainland Australia (Davies et al. 2018; Neave et al. 2024). These processes include predation by feral cats and habitat modification by feral herbivores in combination with frequent high severity fires. In addition, around 30,000 ha of eucalypt open forest habitat on Melville Island has been converted into short‐rotation plantations of *Acacia mangium* (Woinarski 2001).

Although there is high fire frequency across both islands, Bathurst Island benefits from lower feral cat density and the absence of feral herbivores such as Asian water buffalo ( *Bubalus bubalis* ) and horse ( *Equus caballus* ). Bathurst Island is also characterised by relatively uniform and higher annual rainfall (1800–2000 mm), whereas Melville Island has a stronger rainfall gradient (1400–2000 mm), with drier conditions in the eastern portion. The consistently higher rainfall on Bathurst likely promotes greater primary productivity, which likely helps mitigate the threatening processes seen on Melville Island (Stobo‐Wilson et al. 2020a; von Takach et al. 2020).

### 2.2 Study Species

We investigated three co‐occurring species with contrasting resource requirements, demographics, and dispersal abilities: the northern brushtail possum, the northern brown bandicoot and the black‐footed tree‐rat. Northern brushtail possums and black‐footed tree‐rats are arboreal, nesting in tree hollows in the canopy, whilst northern brown bandicoots are ground dwelling and nest in shallow holes, grass and other debris. Black‐footed tree‐rats only occur on Melville Island and rely on a well‐developed mid‐storey shrub layer that tends to be associated with low fire frequencies (Friend 1987). Under Australian national legislation, northern brushtail possums are listed as Vulnerable whilst northern brown bandicoots are not listed. The Tiwi Island subspecies of black‐footed tree‐rat, *M. gouldii melvillensis*, is not listed; however, the two subspecies on the mainland are listed as Vulnerable (von Takach et al. 2020). However, contractions in range and abundance have been reported from Melville Island and mainland Northern Territory for all three species (Davies et al. 2018; von Takach et al. 2020; Neave et al. 2024). All three species fall within the critical weight range of species vulnerable to predation from feral cats (Murphy and Davies 2014).

### 2.3 Data Collection

Tissue samples were collected from Bathurst and Melville Islands during surveys conducted between 2018 and 2023. These surveys were conducted under several research projects and as a result, different survey designs were used. Details of each survey can be seen in their respective study (Carey et al. 2025; Davies et al. 2021; Neave et al. 2024). Live trapping was conducted using cage traps (56 × 20 × 20 cm) and box traps (33 × 10 × 9 cm) with standard mammal bait of oats, peanut butter, and honey. An ear tissue sample was collected from each captured individual for genetic analysis.

### 2.4 Genetic Data

Northern brown bandicoot and northern brushtail possum tissue samples were sent to the Diversity Arrays Technology sequencing (DArTseq) facility (Canberra, Australia) for de novo genotyping of single nucleotide polymorphisms (SNP), as described in Kilian et al. (2012). Before filtering, SNP data sets included 300 unique northern brushtail possum samples genotyped at 69,845 loci, and 192 unique northern brown bandicoot samples genotyped at 57,002 loci. The DNA sequences were subject to further filtering using the ‘dartR’ R package (Gruber et al. 2018) to only include biallelic SNPs and to exclude SNPs with a call rate < 0.8, an allele depth ratio > 2, reproducibility rate < 95%, a read depth < 10×, and sex‐linked SNPs beneath a tolerance threshold of 2.5% female heterozygosity were dropped. Additionally, individuals with a call rate < 0.7 were removed. Filtering for linkage disequilibrium was not conducted; however, secondaries were dropped to remove closely linked SNPs. Black‐footed tree‐rat tissue samples were sent to the Australian Genome Research Facility (AGRF) for genotyping of SNPs; as described in Peterson et al. (2012). The difference in external laboratory was to maintain consistency with previously sequenced black‐footed tree‐rat samples referenced to the *Mastacomys fuscus* genome (as in von Takach et al. 2023). Before filtering, the SNP data set included 160 black‐footed tree‐rat samples genotyped at 961,867 loci. Data for chromosome, SNP position, AlleleID, read depth, and allele depth ratio were extracted from the Variant Call Format (VCF) file before being transferred into ‘dartR’ for filtering consistent with the other species. The number of SNPs resulting from each filtering step that were used for downstream analyses can be found in Table S1.

### 2.5 Genetic Diversity and Structure

Using the ‘dartR’ R package we calculated summary statistics for each sampling site (Figure S1) including observed (*H*_O_) and expected (*H*_E_) heterozygosity, wright's fixation index (*F*_IS_) and genetic differentiation (*F*_ST_). To visualise genetic diversity patterns of each species across the islands, we applied a moving‐window approach with the ‘wingen’ R package (Bishop et al. 2023) whereby *H*_O_ was calculated for each 10 km^2^ window with at least two individuals present. This enabled us to account for variations in local sample sizes by integrating estimates over larger areas. The output was extended to a continuous surface of genetic diversity covering the entirety of the islands by interpolating the data points using empirical Bayesian kriging and masking to the original layer.

Population structure of each species was inferred using principal coordinates analyses (PCoA) in ‘dartR’ and ancestry analyses using the ‘Landscape and Ecological Associations’ (LEA) R package (Frichot and François 2015). The PCoA is a distance‐based approach to dissect and display dissimilarities between individuals. The LEA package calculates ancestry coefficients using the snmf() function with parameters set at *K* = 1–5 and five repetitions. Cross‐entropy criteria were used to select the optimum number of K populations.

To investigate patterns of spatial genetic structure for each species across each island, we used spatial autocorrelation analyses of multilocus genotypes, using ‘GenAlEx’ (Peakall and Smouse 2006). We calculated an overall spatial autocorrelation for each species using distance class boundaries of 10, 20, 30, 40, 50, 60, 70, 80, 90, 100 and 110 km (maximum distance across the study area). The significance of combined $r_{c}$ values was assessed with 1000 permutations and 95% confidence intervals around $r_{c}$ calculated with 1000 bootstrap iterations.

### 2.6 Drivers of Landscape Genetic Patterns

We identified 11 candidate resistance surfaces to determine whether landscape characteristics (fire, rainfall, vegetation, topography, watercourses or interspecies interactions) impede or facilitate gene flow (Table 1, Figure S2a–k). Each resistance layer was aggregated to a resolution of 250 m^2^, as this distance balances ecological meaning and processing power. We then calculated individual‐based genetic distances using Euclidean distance of the first 20 axes of a PCoA, which has been found to maximise accuracy and perform well at low sample sizes (Shirk et al. 2018). The northern brushtail possum data from Bathurst Island had a slight negative relationship between genetic distance and geographic distance, violating the optimisation analysis requirements. We therefore rank‐transformed the genetic data to achieve a slight positive Isolation By Distance (IBD) relationship whilst preserving the relative relationships among genetic distances.

**TABLE 1:** Resistance surfaces included in landscape genetic analysis.

Landscape characteristics	Description	Data type
Cat activity	GLM of cat detections from camera trapping data producing a layer of predicted nightly trap success.	Continuous
Feral herbivore activity	GLM of buffalo and horse detections from camera trapping data producing a layer of predicted nightly trap success.	Continuous
Fire frequency	Number of years burnt between 2000 and 2023. Source: NAFI (2024).	Continuous
Late fire frequency	Number of years burnt after July 31st between 2000 and 2023. Source: NAFI (2024).	Continuous
Time since fire	Number of years since last burnt. Source: NAFI (2024).	Continuous
Vegetation	Type of vegetation class. Used both a simplified layer of four classes and a more complex layer of seven classes.	Categorical (7/4)
Elevation	The height of a cell from sea level. Derived from SRTM, DEM, with a 30‐m resolution. Source: Geoscience Australia (2024b).	Continuous
Ruggedness	Topographical ruggedness calculated from the difference in elevation between a cell and the eight cells surrounding it. Source: Geoscience Australia (2024a).	Continuous
Rainfall	Mean annual rainfall (mm). Source: BIOCLIM (2024).	Continuous
Distance to coast	Distance (m) to coast.	Continuous
Distance to water	Distance (m) to a perennial or non‐perennial watercourse.	Continuous
Geographic distance	Automatically included in ResistanceGA.	Continuous
NULL	Automatically included in ResistanceGA.	N/A

Abbreviations: DEM, digital elevation model; GLM, generalised linear model; SRTM, shuttle radar topography mission.

One of the 11 resistance surfaces comprised categorical data, derived from a *Vegetation* layer representing seven broad vegetation classes across the Tiwi Islands: forests, woodlands, rainforests, plantation forests, treeless plains, salt areas and developed areas. We also included a simplified version of the *Vegetation* layer comprising four main vegetation classes: forests, plantation forests, treeless plains and salt areas. In this layer, the forest category incorporated woodlands, rainforests, and developed areas, as the small, isolated patches of rainforest and developed land may be inconsequential to genetic patterns and thus unnecessarily penalising this layer. We chose the better performing layer to model and interpret. The remaining 10 layers were continuous data with three resistance surfaces representing fire regime patterns. *Fire frequency* and *Time since fire* represented spatiotemporal fire patterns and *Late‐season fire frequency* representing temporal and spatial severity patterns with more intense fires predicted in the late dry season (Russell‐Smith and Edwards 2006). Fire layers were downloaded from the Northern Australia Fire Information (NAFI) website (https://firenorth.org.au/nafi3/) and incorporate data from 2000 to 2023. Mean annual rainfall (BIO12) was included to account for the strong rainfall gradient from east to west and the weaker rainfall gradient from south to north apparent on Bathurst Island. After conducting a PCA of all BIOCLIM variables we decided to use BIO12 individually, as BIO12 was (1) responsible for 95% of the variation across the Tiwi Islands; (2) highly correlated with other BIOCLIM variables and (3) ecologically interpretable. The *Elevation* layer was downloaded from Geoscience Australia along with the topographic *Ruggedness* layer, which was calculated from the difference in elevation between a cell and the eight cells surrounding it. Distance to the coast was calculated from a base map of the Tiwi Islands coastline using the ‘terra’ R package (Hijmans et al. 2022). Distance to water was similarly calculated from a layer of both perennial and non‐perennial rivers, creeks, and streams across the Tiwi Islands. Two continuous layers of feral species were also included. Feral cat and feral herbivore activity was calculated using nightly detections from a long‐term camera trapping dataset across the Tiwi Islands (Davies et al. 2020; Neave et al. 2024). Cat, and buffalo and horse detections were used in multiple Generalised Linear Models (GLM) incorporating geographic position, survey type and autocovariates. The top model was chosen based on Akaike Information Criterion (AIC) corrected for finite sample size (AICc) and used it to map cat and herbivore activity. To improve accuracy, herbivore camera detections were supplemented by expert knowledge from aerial surveys across the islands. All 11 layers were inspected for correlations using the layerCor() function in the ‘terra’ R package to ensure < 0.8 Pearson's correlation coefficient (Table S3). The highest correlation was between the distance to coast and elevation layers (*r* = 0.73), followed by the correlation between time since fire and fire frequency (*r* = −0.58). Finally, geographic distance to test for IBD and a null model without any landscape structure (panmixia) were automatically included as alternative models by the ‘ResistanceGA’ R package (Peterman 2018).

To determine which landscape features or combination of landscape features best explained the observed genetic structure, we used the ‘ResistanceGA’ R package. ‘ResistanceGA’ implements nonlinear transformations to adaptively explore and improve the relationship between the matrices of pairwise resistance distance and genetic distance. It fits linear mixed‐effects models with a Maximum Likelihood Population Effects (MLPE) parameterisation (Clarke et al. 2002) and uses the log‐likelihood to optimise the relationship. Resistance distances were calculated in Circuitscape (McRae et al. 2008) implemented through the Julia programming language (v. 1.14.9, accessed at https://julialang.org/). We first optimised each surface individually and inspected the model results based on AICc. Layers that performed better than the IBD and null models were then considered in the multivariate analysis and were combined (up to a maximum of two) to assess whether multiple surfaces better explained the observed genetic structure patterns. As analyses were conducted for three species across one or two islands and at two spatial scales (10 datasets in total), combined resistance surfaces were limited to a maximum of two layers to balance computational feasibility with the ability to evaluate a broad and comparable set of landscape hypotheses across species, islands and scales.

As a measure of uncertainty and to assess model stability, we refit the models across 1000 bootstrap iterations of randomly subset individuals (75% of total samples) and recorded the percentage of iterations each surface was the top‐ranked model, based on AIC adjusted for small‐sample sizes (AICc, Anderson and Burnham 2002).

### 2.7 Fine‐Scale Landscape Drivers of Genetic Patterns

The above analysis identifies the landscape drivers of genetic patterns across each entire island, a broad spatiotemporal scale. To identify the landscape drivers on a scale relevant to dispersal events between survey sites and within habitable savanna, we used the results of the spatial autocorrelation analysis to set a threshold of > 250 and < 20 km. By using a matrix of ‘pairs to include’ in ‘ResistanceGA’, we indicated which pairwise comparisons should be included when optimising resistance values at a fine scale.

## 3 Results

### 3.1 Genetic Data Sets

After filtering, the final SNP data sets included 291 northern brushtail possum samples (162 Melville, 129 Bathurst) genotyped at 19,397 loci, 183 northern brown bandicoot samples (139 Melville, 44 Bathurst) genotyped at 8265 loci, and 142 black‐footed tree‐rat samples genotyped at 14,553 loci (Table S1). We then removed one relative from each identified pair resulting in a final data set of 228 northern brushtail possum (122 Melville, 106 Bathurst), 152 northern brown bandicoot (129 Melville, 23 Bathurst), and 110 black‐footed tree‐rat (only on Melville). As some sites had small sample sizes (*n* = 3), *H*_O_, *F*_IS_ and *F*_ST_ estimates may be imprecise. Therefore, these values are presented for descriptive comparison only and were not used in subsequent individual‐based analyses.

### 3.2 Genetic Diversity Patterns

**FIGURE 2:**
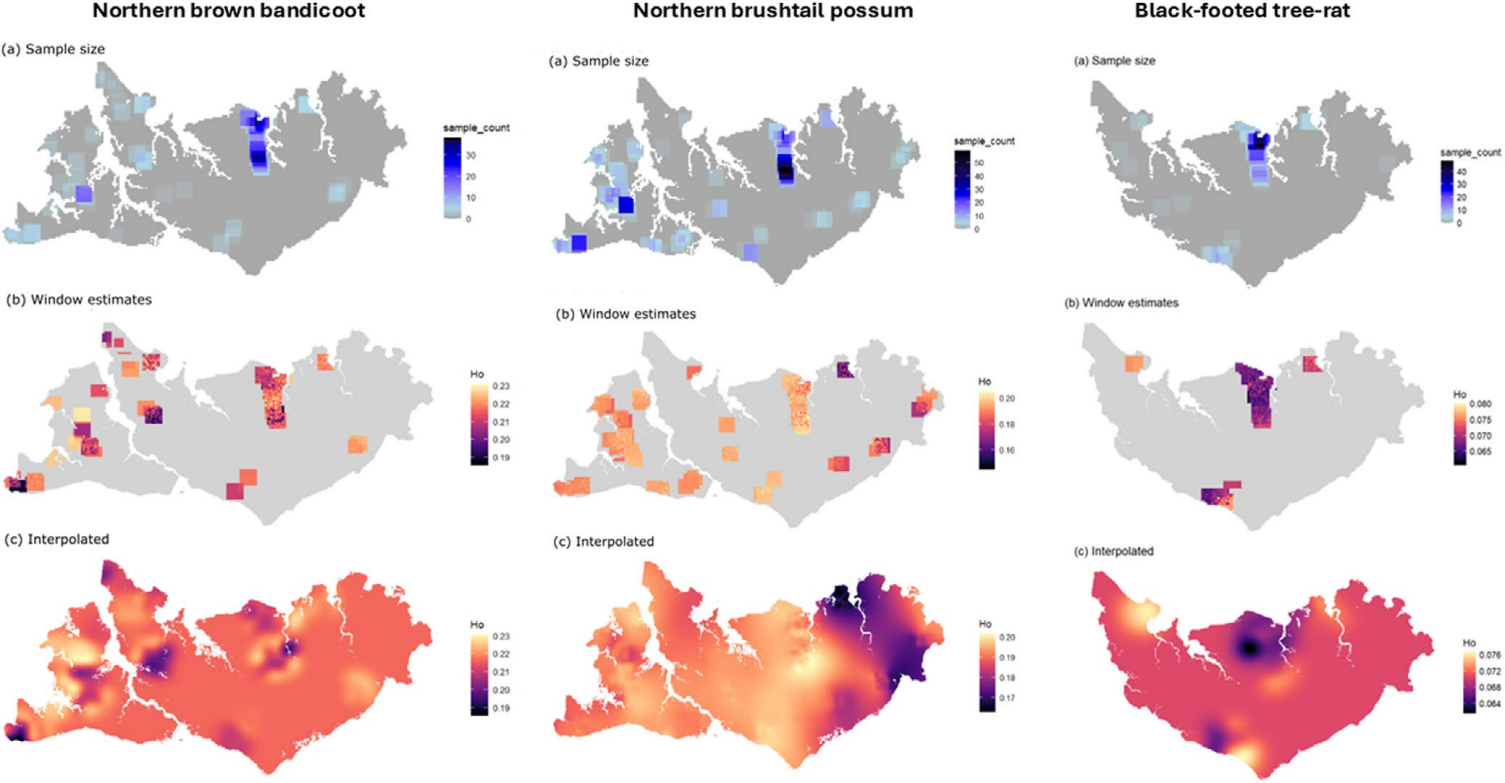
Moving window estimates for each species of (a) sample size, (b) observed heterozygosity, and (c) observed heterozygosity interpolated across the landscape.

Observed heterozygosity (*H*_O_) for the northern brushtail possum ranged from 0.16 to 0.20 on Melville Island but was consistently around 0.19 on Bathurst Island. For northern brown bandicoots, *H*_O_ ranged from 0.18 to 0.23 and 0.18 to 0.22 on Melville Island and Bathurst Island, respectively. In contrast, black‐footed tree‐rats, *H*_O_ was substantially lower, ranging from 0.068 to 0.078 on Melville Island, with significantly higher *F*_IS_ values (Table 2, Table S2). The moving window estimates of genetic diversity (*H*_O_) and the interpolation of these estimates reveal the spatial variation between species and islands (Figure 2).

**TABLE 2:** Sample size and genetic diversity estimates including observed heterozygosity ($H_{O}$), unbiased expected heterozygosity (${\mathrm{uH}}_{E}$), and wright's fixation index (*F*_IS_) calculated across Bathurst and Melville Islands for each species.

Species	Island	*n*	*H*_O_	*H*_O_ range	*uH*_E_	*uH*_E_ range	*F*_IS_	*F*_IS_ range
Northern brushtail possum	Bathurst	104	0.192	0.187–0.195	0.229	0.221–0.234	0.161	0.133–0.184
	Melville	116	0.191	0.166–0.197	0.227	0.216–0.243	0.156	0.150–0.235
Northern brown bandicoot	Bathurst	13	0.192	0.191–0.193	0.234	0.226–0.243	0.180	0.154–0.205
	Melville	111	0.220	0.202–0.226	0.262	0.214	0.160	0.142–0.206
Black‐footed tree‐rat	Melville	108	0.070	0.068–0.076	0.248	0.211–0.265	0.714	0.640–0.745

### 3.3 Genetic Structure Patterns

**FIGURE 3:**
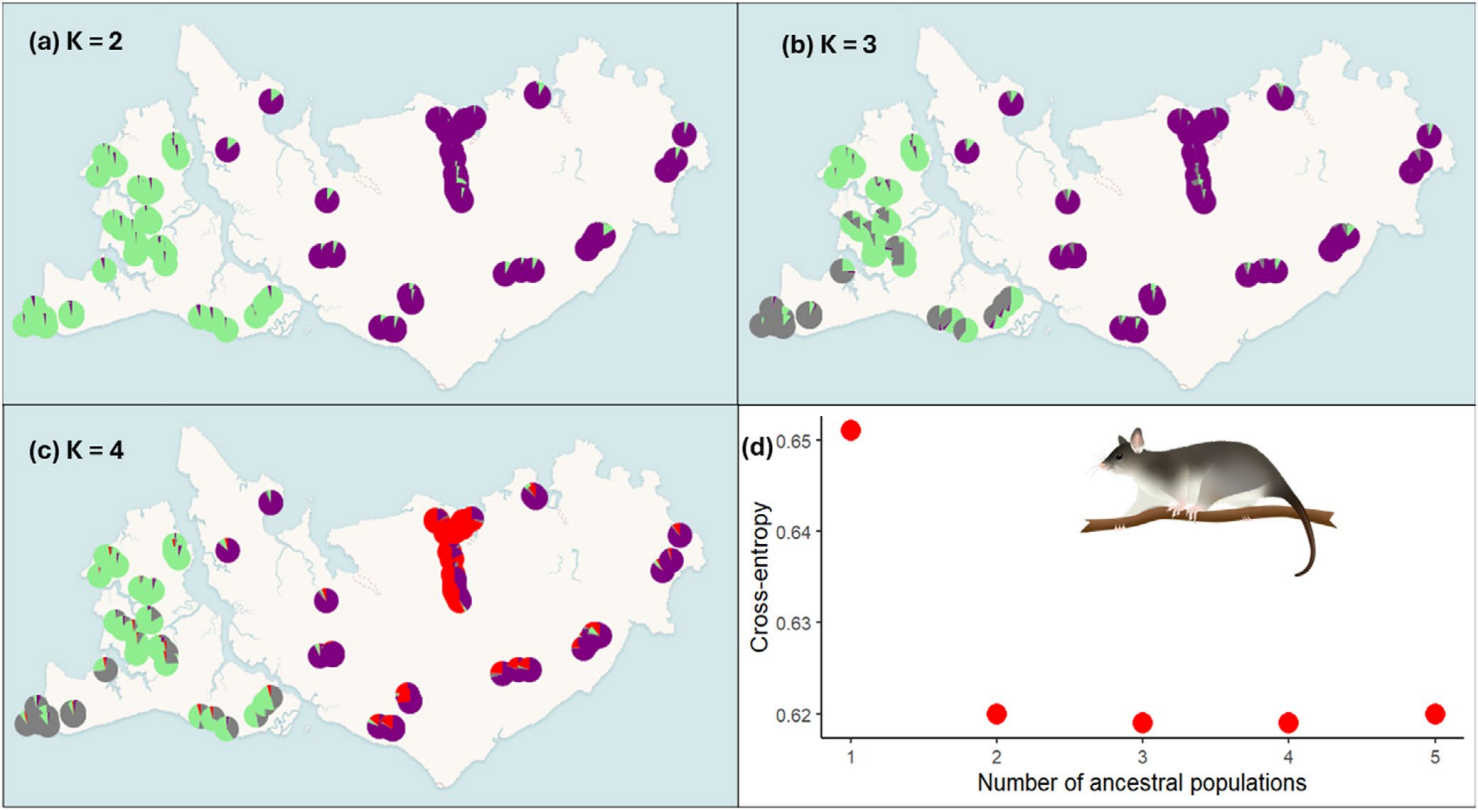
Patterns of landscape genetic structure across the Tiwi Islands for the northern brushtail possum. Mean ancestry proportions for (a) *K* = 2, (b) *K* = 3, and (c) *K* = 4, calculated using LEA. (d) cross‐entropy scores for *K* = 1–5, where the lowest value indicates the best prediction of genetic ancestry. The large drop in cross‐entropy between 1 and 2 ancestral populations indicates that *K* = 2 is well‐supported.

For northern brushtail possums, *F*_ST_ ranged from 0.0001 to 0.0480 on Bathurst Island and 0.005 to 0.049 on Melville Island (Tables S4 and S5). Genetic structure analysis revealed two genetic clusters, one for each island (Figure 3a,d). When visualising three genetic clusters, Bathurst Island separates into two clusters, north and south (Figure 3b). When visualising four genetic clusters, the central northern populations on Melville Island became a separate cluster (Figure 3c). Similarly, the PCoA analysis demonstrated that there was more genetic structure on Bathurst Island than Melville Island (Figure S3a); however, the distinct central northern cluster could be detected when viewing Melville Island separately (Figure S4a).

**FIGURE 4:**
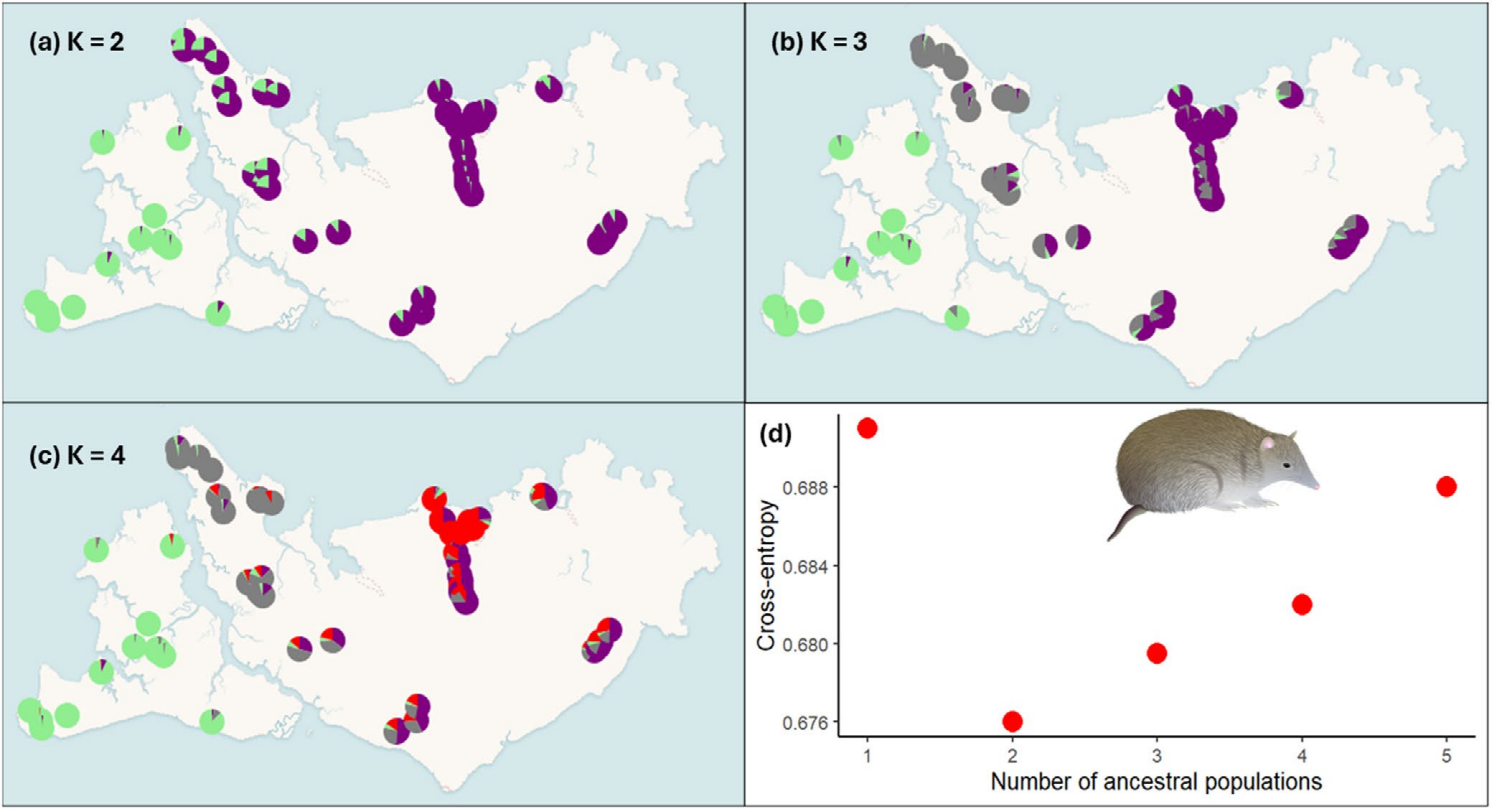
Patterns of landscape genetic structure across the Tiwi Islands for the northern brown bandicoot. Mean ancestry proportions for (a) *K* = 2, (b) *K* = 3, and (c) *K* = 4, calculated using LEA. (d) cross‐entropy scores for *K* = 1–5, where lowest value indicates the best prediction of genetic ancestry. The large drop in cross‐entropy between 1 and 2 ancestral populations indicates that *K* = 2 is well‐supported.

For northern brown bandicoots, pairwise population *F*_ST_ was 0.038 on Bathurst Island and ranged from 0.006 to 0.056 on Melville Island (Tables S6 and S7). Genetic structure analyses revealed two genetic clusters, one on Bathurst Island, which is also present in northwest Melville Island, and another on Melville Island (Figure 4a,d). When visualising three genetic clusters, the northwest Melville populations become a distinct cluster (Figure 4b). When visualising four genetic clusters, Melville Island separated into three clusters whilst Bathurst Island remained at one (Figure 4c). The PCoA analysis supports this stronger genetic structure on Melville Island and more panmictic pattern on Bathurst Island (Figure S3b) with clear differentiation between the northwest and the rest of Melville Island (Figure S4b).

**FIGURE 5:**
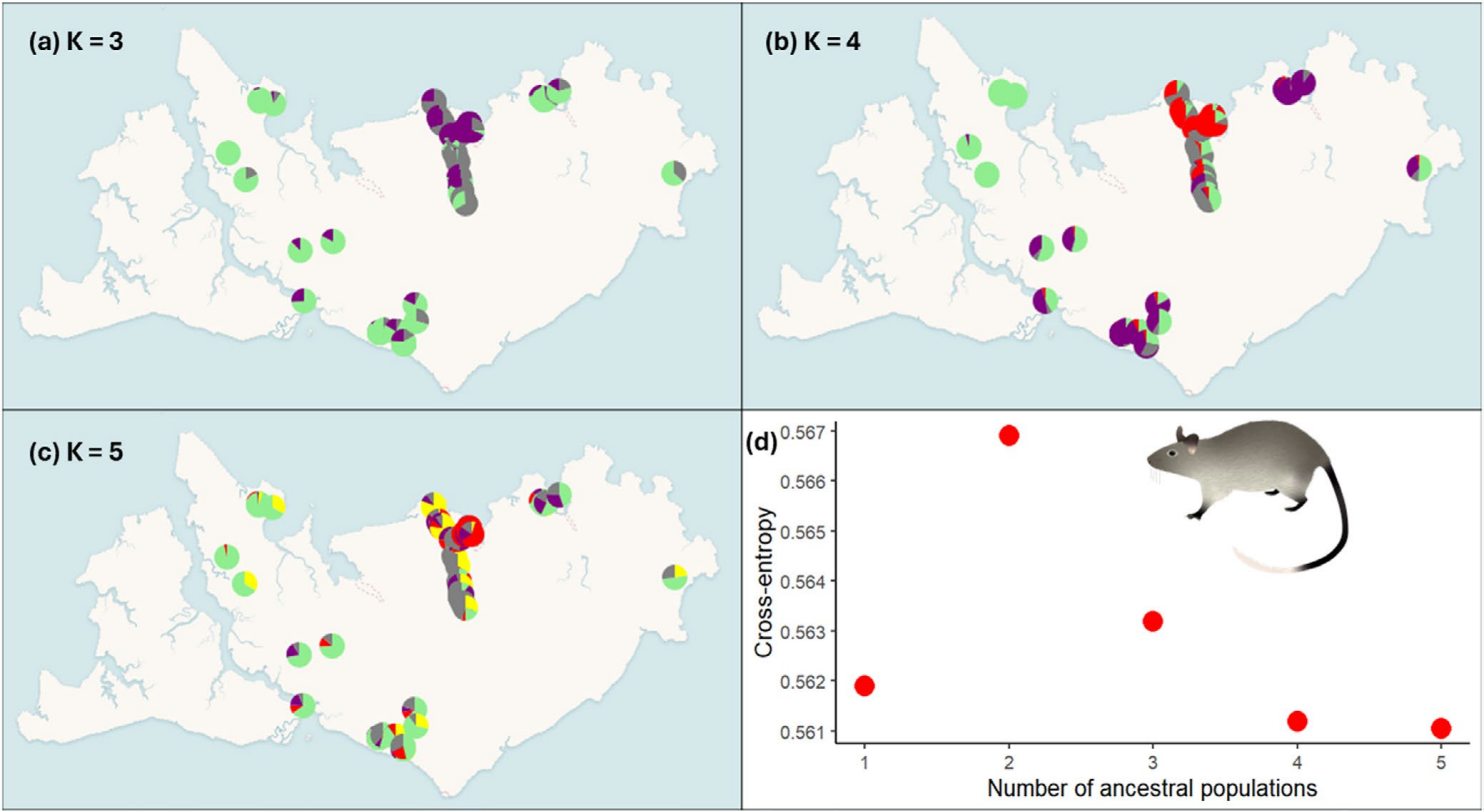
Patterns of landscape genetic structure across the Tiwi Islands for the black‐footed tree‐rat. Mean ancestry proportions for (a) *K* = 3, (b) *K* = 4, and (c) *K* = 5, calculated using LEA. (d) cross‐entropy scores for *K* = 1–5, where lowest value indicates the best prediction of genetic ancestry. The large drop in cross‐entropy between 2 and 3 ancestral populations indicates that *K* = 3 is well‐supported, followed by *K* = 4.

For black‐footed tree‐rats, pairwise population *F*_ST_ ranged from 0.002 to 0.047 (Table S8). Three to five clusters best represented the genetic data (Figure 5d). Three genetic clusters revealed that the east and west populations were more similar and that there were two separate clusters in the central northern populations (Figure 5a). When visualising four and five genetic clusters, the genetic structure of the central northern populations increased (Figure 5b,c). The PCoA analysis demonstrated the greater similarity between eastern and western populations and the distinction and high variability within the central populations (Figure S4c).

### 3.4 Spatial Autocorrelation

**FIGURE 6:**
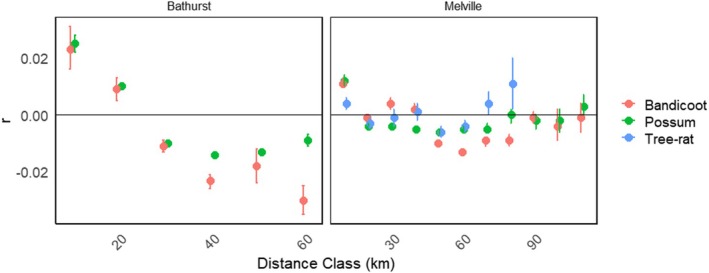
Spatial autocorrelation analysis for northern brown bandicoots, northern brushtail possums and black‐footed tree‐rats across Bathurst and Melville Island. Correlation estimates are calculated for each distance class and plotted at the endpoint with 95% bootstrap confidence intervals based on 1000 permutations.

Spatial genetic structure on Bathurst Island revealed similar patterns between northern brown bandicoots and northern brushtail possums up to ~30 km (Figure 6). At greater distances (40–60 km), northern brown bandicoots became more negatively autocorrelated than northern brushtail possums. On Melville Island, spatial genetic structure patterns were more variable between species. Northern brushtail possums followed a similar trajectory as on Bathurst Island, whilst northern brown bandicoots, despite an initial drop, remained positively correlated until ~40 km. Northern brown bandicoots were more negatively autocorrelated than the other species between 40 and 80 km. Both northern brushtail possums and northern brown bandicoots were randomly correlated by 90 km. Black‐footed tree‐rats showed little spatial genetic structure at distances up to 40 km, but were negatively correlated between 50 and 60 km. However, between 70 and 80 km, they were positively correlated reflecting the genetic similarity between east and west Melville populations, as shown in Figure 5.

### 3.5 Landscape Drivers of Genetic Patterns at Fine and Broad Scales

On Bathurst Island, AICc ranked modelling identified a clear and single landscape driver for both northern brushtail possums and northern brown bandicoots. At the broad scale, higher rainfall in the north was associated with high resistance to gene flow for northern brushtail possums, selected as the top‐ranked model in 99.7% of iterations. For northern brown bandicoots, geographic distance (IBD) was selected as the top‐ranked model in 96.9% of iterations (Table 3). At a fine scale, the drivers of genetic patterns on Bathurst Island were similar for northern brushtail possums with high rainfall as the top‐ranked model (61.9% of iterations; Table 3, Figure S5). For northern brown bandicoots, the high fire frequency in the central area of Bathurst Island was associated with high resistance to gene flow at the fine scale (71% of iterations; Table 3, Figure S6).

**TABLE 3:** Model selection results for the top five environmental attributes that best explain broad scale (all samples) and fine scale (< 20 km) genetic structure patterns on Bathurst Island. Results were aggregated over 1000 bootstrap iterations and sorted by average rank.

Species	Broad‐scale surface				Fine‐scale surface			
		Average rank	% top‐ranked	Average ∆ AICc		Average rank	% top‐ranked	Average ∆ AICc
Northern brushtail possum	Rainfall	1.00	99.7	0	Rainfall	2.3	61.9	0
	Distance to coast and rainfall	2.51	0.0	7.25	Late fire frequency	4.7	12.3	3.60
	Rainfall and ruggedness	2.86	0.3	11.15	Distance to coast	4.80	8.4	3.51
	Rainfall and distance to water	4.13	0	12.59	Geographic distance	5.30	8.8	3.50
	Fire frequency and rainfall	5.32	0	13.08	Distance to coast and rainfall	6.67	1.6	5.58
	Elevation and rainfall	5.57	0	13.25	Ruggedness	6.69	3.9	4.98
Northern brown bandicoot	Geographic distance	1.04	96.9	0	Fire frequency	1.73	71	0
	Distance to water	3.45	0.2	5.60	Late fire frequency	5.63	6.2	5.90
	Rainfall	4.56	0.0	6.54	Distance to coast and fire frequency	5.68	0.1	14.00
	Panmictic (NULL)	4.58	2.9	5.99	Fire frequency and late fire frequency	5.75	0.2	14.05
	Fire frequency	5.68	0	6.79	Distance to coast	5.83	6.8	5.87

Abbreviation: AICc, corrected Akaike information criterion.

On Melville Island, the landscape drivers of broad‐scale genetic patterns were less clear for each species, but dominant drivers could be elucidated. For northern brushtail possums, the composite surface of fire frequency and ruggedness was the top‐ranked model in 37.8% of iterations (Figure S7) followed by late fire frequency and ruggedness in 19.1% of iterations (ΔAICc = 3.65; Table 4). Together, this constitutes 56.9% of iterations. Ruggedness, where high topographic ruggedness was associated with less resistance to gene flow, contributed the majority to both composite surfaces with 59% and 84%, respectively. Areas of low fire frequency were associated with increased resistance to gene flow. However, the broad‐scale analysis included pairwise comparisons across the entire island and therefore accounted for unsuitable habitat areas such as mangroves where no fires had burnt. The increased frequency of late dry season fires was associated with increased resistance to gene flow. At a fine scale, the top‐ranked model explaining the genetic structure patterns of northern brushtail possums on Melville Island was geographic distance (31.6% of iterations). However, the following three surfaces of elevation (27.1% of iterations; Figure S8), time since fire (9.8% of iterations), and topographic ruggedness (22.1% of iterations) had ΔAICc < 2.

**TABLE 4:** Model selection results for the top five environmental attributes that best explain broad scale (all samples) and fine scale (< 20 km) genetic structure patterns on Melville Island.

Species	Broad‐scale surface				Fine‐scale surface			
		Average rank	% top‐ranked	Average ∆ AICc		Average rank	% top‐ranked	Average ∆ AICc
Northern brushtail possum	Fire frequency and ruggedness	3.21	37.8	0	Geographic distance	2.33	31.6	0
	Late fire frequency and ruggedness	4.66	19.1	3.65	Elevation	2.78	27.1	0.78
	Ruggedness and vegetation	5.63	4	9.75	Time since fire	3.16	9.8	1.66
	Fire frequency	5.67	9.3	14.21	Ruggedness	3.51	22.1	1.89
	Ruggedness and time since fire	5.69	1.2	6.90	Elevation and ruggedness	4.44	9	4.81
Northern brown bandicoot	Ruggedness	1.83	76.9	0	Geographic distance	1.50	59.3	0
	Vegetation	3.71	7	13.72	Rainfall	1.92	37.2	1.00
	Fire frequency and vegetation	4.47	8.3	16.14	Ruggedness	3.36	0	4.11
	Ruggedness and vegetation	4.62	2.8	16.30	Fire frequency	4.75	0.9	7.19
	Feral herbivore activity and ruggedness	4.63	2.1	15.17	Rainfall and ruggedness	6.15	1.9	10.15
Black‐footed tree‐rat	Fire frequency	2.02	58.4	0	Rainfall and ruggedness	2.29	45.8	0
	Cat activity and fire frequency	6.65	1.2	5.91	Ruggedness and vegetation	3.75	17.3	4.56
	Feral herbivore activity	6.93	3.5	4.64	Fire frequency and ruggedness	4.29	30.4	4.32
	Cat activity and vegetation	7.27	11.9	10.03	Fire frequency	6.28	0.9	8.40
	Cat activity	7.36	0.4	4.87	Fire frequency and rainfall	6.36	0.6	10.02

*Note:* Results were aggregated over 1000 bootstrap iterations and sorted by average rank.

Abbreviation: AICc, corrected Akaike information criterion.

For northern brown bandicoots, broad‐scale genetic structure patterns on Melville Island were best explained by topographic ruggedness, the top‐selected model in 76.9% of iterations (Table 4). Similarly to northern brushtail possums, high topographic ruggedness was associated with less resistance to gene flow (Figure S9). Vegetation had the second‐best average rank with the categories of mangroves and plantation associated with high resistance to gene flow. Whilst this model is much less supported (ΔAICc = 13.72), these vegetation types are dominant in the north‐west peninsula of Melville Island, and this resistance perhaps explains the reduced genetic diversity (Figure 2) and increased structure patterns (Figure 4a). At a fine scale, geographic distance best explained the genetic structure patterns of northern brown bandicoots on Melville Island (59.3% of iterations) followed by rainfall (37.2% of iterations, ΔAICc = 1; Table 4) with high rainfall in the north‐west conferring high resistance to gene flow (Figure S10).

For black‐footed tree‐rats, fire frequency was the top‐ranked model at the broad scale with areas of low‐fire frequency associated with high resistance to gene flow (58.4% of iterations; Figure S11). At a fine scale, the composite surface of topographic ruggedness and rainfall was the top‐ranked model with high topographic ruggedness and low rainfall associated with high resistance to gene flow (45.8% of iterations; Figure S12). Topographic ruggedness was selected in the top three models in combination with rainfall, vegetation and fire frequency, respectively (Table 4). Low rainfall areas of eastern Melville, less suitable vegetation such as mangroves and treeless plains, and low fire frequencies (burnt < 5 of 22 years) were associated with high resistance to gene flow. All areas burnt > 5 times in 22 years were of low resistance at a fine scale. This highlights that only plantation and mangrove habitat are resistant to gene flow between populations, whilst habitable savanna landscapes are of low resistance to gene flow.

## 4 Discussion

We found evidence for species‐, island‐, and scale‐specific drivers of landscape genetic connectivity. On Bathurst Island, where threatening processes are relatively benign and rainfall is high, the more dispersive and ground‐dwelling northern brown bandicoot exhibited less genetic structure (Figure 4) than the more sedentary and arboreal northern brushtail possum (Figure 3). In contrast, on Melville Island the pattern for these same species was reversed, while the habitat specialist black‐footed tree‐rat showed the lowest genetic diversity (Figure 2) and the strongest genetic structure (Figure 5) of all three species. On Bathurst Island, drivers of genetic structure were readily identified but differed between species (Table 3), likely reflecting differences in life‐history traits. Drivers of genetic structure on Melville Island were not directly associated with frequent fire, feral cats or feral herbivores, as hypothesised, but were instead commonly associated with high topographic ruggedness or uninhabitable areas of low fire frequency (Table 4). Our hypothesis that broad‐scale analyses would detect stable landscape features and fine‐scale analyses would detect dynamic disturbances was only partially supported. Restricting the fine‐scale analysis to savanna habitat, however, was essential for identifying fire‐related reductions in connectivity relevant to management. The influence of fire in shaping genetic connectivity within savanna (i.e., at the fine spatial scales) was more apparent on Bathurst Island, suggesting fire management may be an effective strategy in this landscape. In landscapes subject to additional threatening process; however, fire management needs to be supported by broader conservation actions to mitigate those additional threats.

### 4.1 The Role of Fire in Shaping Genetic Connectivity

Landscape genetic techniques have been applied to conservation planning to identify the impacts of contemporary issues such as habitat fragmentation (Thatte et al. 2020), feral species (Mothes and Searcy 2024) and climate change (Maier et al. 2022). Despite fire regimes being altered across the globe, with increasing size, frequency and intensity (Sayedi et al. 2024), fire has rarely been incorporated into landscape genetic studies (Pereoglou et al. 2013; Ruiz‐Lopez et al. 2016; Shaw et al. 2023). Yet, by shaping habitat dynamics and resource availability, fire can influence dispersal and reproduction, ultimately contributing to the spatial structuring of genetic diversity within and among populations (Banks et al. 2013). A key challenge of incorporating dynamic variables such as fire into landscape genetic analyses is that observed genetic patterns reflect historical gene flow, resulting in a temporal lag between actual gene flow events and measurable genetic signals (Landguth et al. 2010). However, the availability of long‐term, high‐quality, satellite‐derived fire data allowed us to incorporate fire history from the preceding 22 years, a relatively long period in this frequently burnt system. Nevertheless, the fine spatiotemporal effects of fire on populations make it difficult to interpret broad‐scale landscape genetic data and is a potential limitation of this study. Another complexity for studying fire‐genetic associations is that environmental drivers of gene flow may act at different spatial scales within a species and vary between species due to differences in dispersal capacity (Cushman and Landguth 2010). By incorporating multiple species at both the empirically derived fine scale (20 km) and the broad (whole island) scale, our study was well positioned to detect scale and species‐specific effects.

Fire is a conservation concern across northern Australia, with evidence that declines in small mammal species is heightened in areas with larger and more frequent fires and reduced availability of long‐unburnt vegetation (Einoder et al. 2023). However, the influence of fire on connectivity between populations has not been examined in this landscape and may reveal a mechanism of isolation. In this study, the role of fire in shaping genetic patterns within savanna habitat (i.e., at a fine spatial scale) was only detected for one species, the northern brown bandicoot, and only on Bathurst Island. Differences in life history likely explain these contrasting responses, with frequent fire influencing the genetic structure of the ground‐dwelling, more dispersive northern brown bandicoot, but not the arboreal and more sedentary northern brushtail possum. Bathurst Island has been found to support healthy populations of both species (Neave et al. 2024), including in areas of high fire frequency (Davies et al. 2021). High resistance to gene flow was only apparent in areas burnt > 20 times in 22 years (Figure S6), suggesting that very high fire frequencies (or fire return intervals), rather than moderate fire frequencies may constrain population connectivity. In this system, reducing fire frequency from near‐annual burning to approximately once every 1.3 years may effectively improve genetic connectivity. Frequent late dry‐season fire was also the second‐ranked model for both species at the fine scale, although ΔAICc > 2, indicating weaker support.

For northern brushtail possums, reduced connectivity was instead associated with high rainfall at both spatial scales. Higher rainfall may increase resource availability and reduce the need for dispersal, thereby strengthening local genetic structure. Bathurst Island supports high densities of northern brushtail possums (Davies et al. 2020), and theoretical models show that strong genetic structure can emerge in high‐density systems when dispersal distances are short (Rousset 2000).

On Melville Island, the role of fire in shaping genetic structure was at a broad, whole island scale where low fire frequency reduced genetic connectivity between northern brushtail possums and black‐footed tree‐rats. However, the highest resistance to gene flow was driven by areas burnt < 5 times in 22 years, such as mangroves and plantations (Figures S7c and S11b), suggesting habitat suitability is the underlying driver. Instead of a direct influence of fire, other climatic, vegetation or topographic factors were commonly identified as drivers of genetic connectivity and are discussed below.

### 4.2 Landscapes With Multiple Interacting Threats

The combined influences of fire and other threats on genetic connectivity have rarely been studied (Sitters and Di Stefano 2020; Tulloch et al. 2016). On Melville Island, the drivers of genetic structure were less clear, with many cases of top models receiving a high mean rank and low percent top rank, and with multiple models with ΔAICc < 2. Our hypothesis that genetic structure on Melville Island would be driven by threats such as frequent fire, increased feral cat activity, and increased feral herbivore activity received no support. Predation by feral cats has been identified as the primary driver of small mammal declines on Melville Island (Davies et al. 2018; Neave et al. 2024) and across northern Australia more broadly (Woinarski et al. 2011). Likewise, the exacerbating effects of feral herbivores have been demonstrated (Legge et al. 2011). Our findings do not contradict the role these feral species play in small mammal declines; rather, they suggest that habitat suitability driven by fire frequency and topographic ruggedness has a stronger, more detectable influence on genetic connectivity at the broad scale. Other studies have demonstrated that spatial patterns of mortality risk can strongly influence landscape genetic connectivity (Ash et al. 2020), and that reductions in apex predators can facilitate the gene flow of mesopredators (Beer et al. 2024). In our study, feral cat and herbivore activity layers were derived from camera detections and GLMs to spatially predict nightly camera‐trap success. As we continue to improve our estimates of feral cat and herbivore abundance and occurrence using GPS collars and aerial count data, we will be better able to resolve their potential influence on the genetic connectivity of native mammals.

A more typical use of landscape genetic techniques is to determine how habitat fragmentation influences genetic connectivity (Balkenhol et al. 2016; Storfer et al. 2010). Vast tracts of savannas on Melville Island have been removed for the establishment of exotic *A. mangium* plantations (Woinarski 2001). These plantations occur predominantly on the western half of Melville Island, but there has been little research done on the direct effects of these plantations on small mammals. In this study, we find the impact of vegetation type on genetic connectivity is evidenced through the high resistance to gene flow of the very low fire frequency areas such as mangroves and plantations on all species on Melville Island. The north‐west of Melville Island has a high density of plantations, mangroves, and treeless plains (Figure S2f) that may constitute a ‘choke point’ of gene flow, thereby leading to genetic differentiation as evident in northern brown bandicoot genetic diversity (Figure 2c) and structure patterns (Figure 4a).

The importance of topographic ruggedness for small mammal conservation has been demonstrated elsewhere in northern Australia (McDonald et al. 2020; Moore et al. 2019). Further, topographic roughness has been demonstrated to influence spatial patterns of fire frequency and the resulting genetic structure (Banks et al. 2017). Here, we found that on Melville Island, high topographic ruggedness facilitated gene flow in northern brushtail possums and northern brown bandicoots at the broad scale, which may be reflecting the role of habitat complexity in reducing threats. However, at the fine scale, high topographic ruggedness was of high resistance to gene flow in black‐footed tree‐rats, in combination with low rainfall. Due to the generally low topographic ruggedness of Melville Island, it is likely that it is the fine scale spatial complexity of this layer that associates it with the complex spatial genetic structure of these species in this landscape. The selection of this spatially complex layer as the top‐ranked model potentially reflects the limitations of landscape genetic data in dynamic environments.

### 4.3 Implications for Conservation Management

Conservation management in landscapes where entire taxa are threatened requires balancing the need for broadscale, multi‐species management with localised and species‐specific information (Boyd et al. 2008; Rattis et al. 2018). Fire management is recommended as an effective way of providing conservation across large areas (Driscoll et al. 2010). However, it needs to be guided by an understanding of the mechanisms facilitating species persistence, such as the landscape variables influencing genetic connectivity (Sitters and Di Stefano 2020). A key knowledge gap in fire management for biodiversity conservation identified in Driscoll et al. (2010) was ‘…how factors such as herbivory, predation, fragmentation, invasive species, and weather interact with fire to alter species' responses to fire directly, or via changes to the fire regime’ (p. 1935). Modelling combinations of these landscape variables against spatial‐genetic data offers a way to fill this knowledge gap and inform management decisions.

No single variable was identified as the most influential driver of genetic connectivity across species, islands, and scales. However, on Bathurst Island, clear drivers could be identified including high fire frequency for northern brown bandicoots. In this landscape with high rainfall and minimal impact of threats such as feral cats and herbivores, using fire management to reduce high fire frequency, alongside preventative conservation efforts, may be an effective conservation strategy. On Melville Island, drivers of genetic structure were less clear, with multiple models receiving support. In this landscape of multiple interacting threats, where native mammal populations are declining, a broader strategy that includes feral species control, habitat protection, and fire management is recommended.

## 5 Conclusions

These results demonstrate that fire regimes can influence landscape resistance to gene flow, and that these effects vary among species and can shift within species under different environmental conditions. Modelling genetic connectivity in fire‐prone landscapes without considering the influence of fire is therefore likely to lead to inaccuracies. With high quality long term spatial fire data now readily available, we recommend landscape genetic studies incorporate fire regimes to better inform context‐specific conservation management.

## Author Contributions

**Alexander R. Carey:** conceptualization (lead), data curation (lead), formal analysis (lead), methodology (lead), writing – original draft (lead). **Teigan Cremona:** conceptualization (equal), methodology (equal), supervision (equal), writing – review and editing (equal). **Georgina Neave:** conceptualization (equal), methodology (equal), writing – review and editing (equal). **Hugh F. Davies:** conceptualization (equal), methodology (equal), supervision (equal), writing – review and editing (equal). **Brett P. Murphy:** conceptualization (equal), methodology (equal), supervision (equal), writing – review and editing (equal). **Geoffrey J. Cary:** conceptualization (equal), methodology (equal), supervision (equal), writing – review and editing (equal). **Tiwi Rangers:** methodology (equal). **Sam C. Banks:** conceptualization (equal), funding acquisition (equal), methodology (equal), supervision (equal), writing – review and editing (equal).

## Funding

This study was funded by the Australian Research Council (ARC) Discovery Project grant (DP210103227), the Indigenous Land and Sea Council, and the ARC Linkage grant (LP170100305) and partially supported by Territory Natural Resource Management (RLPMU03RP11). A. Carey and G. Neave were supported by a Research Training Programme Stipend Scholarship, and a Holsworth wildlife research endowment grant. This study was conducted under the Charles Darwin University Animal Ethics Permit A22010 and the Tiwi Land Council research permit NRM015.

## Conflicts of Interest

The authors declare no conflicts of interest.

## Supporting information


**Data S1:** ece373320‐sup‐0001‐73320.docx.

## Data Availability

The data used in this publication is available online (https://zenodo.org/records/16598763). To use the data, a request must be made for the permission of the Tiwi Land Council.
