# Supplementary material for: The Contrasting Role of Fire in Shaping Landscape Genetic Patterns of Small Mammals Across Two Islands
Source: Ecol Evol. 2026 Mar 26;16(4):e73320. doi: 10.1002/ece3.73320 (PMC13107275; doi:10.1002/ece3.73320)
Supplement: Supplementary file 1 — Data S1: ece373320‐sup‐0001‐73320.docx. [file ECE3-16-e73320-s001.docx]

**The contrasting role of fire in shaping landscape genetic patterns of small mammals across two islands**

Alexander R. Carey^1^, Teigan Cremona^1^, Georgina Neave^1^, Hugh F. Davies^1,2^, Brett P. Murphy^1^, Geoffrey J. Cary^3^, Tiwi Rangers^4^, Sam C. Banks^1^

^1^ Research Institute for the Environment and Livelihoods, Faculty of Science and Technology, Charles Darwin University, Casuarina, NT, 0810, Australia

^2^ School of Environmental and Rural Science, University of New England, Armidale, NSW, Australia.

^3^ Fenner School of Environment & Society, The Australian National University, Canberra, ACT, 2600, Australia

^4^ Tiwi Resources Pty Ltd, Casuarina, NT 0811, Australia.

Corresponding author: Alex Carey, Research Institute for the Environment and Livelihoods, Faculty of Science and Technology, Charles Darwin University, Casuarina, NT, 0810, Australia, [Alex.Carey@cdu.edu.au](mailto:Alex.Carey@cdu.edu.au)

**Supplementary material**

**Tables**

Table S1: Single Nucleotide Polymorphism (SNP) filtering table.

| **Metric** | **Threshold** | **Northern brushtail possum** | **Northern brown bandicoot** | **Black-footed tree-rat** |
| --- | --- | --- | --- | --- |
| Original No. SNPs |  | 69,845 | 57,002 | 961,867 |
| SNP call rate | <0.3 | 67,720 | 54,289 | 218,350 |
| Sex-linked SNPs | <2.5% | 59,981 | 33,423 | 63,636 |
| SNP call rate | <0.8 | 45,925 | 22,571 | 41,750 |
| Reproducibility rate | <95% | 43,477 | 21,927 | NA |
| Read depth | >10x | 27,691 | 10,210 | 41,750 |
| Allele depth ratio | >2 | 24,559 | 9,182 | 10,751 |
| Secondaries | Drop all | 19,397 | 8,265 | NA |

Table S2: Sample size and genetic diversity estimates including observed heterozygosity (*H_O_*), unbiased expected heterozygosity (*uH_E_*), and the fixation index (*F_IS_*) for sampling sites where *n ≥* 3 across Bathurst and Melville Island for each species.

| **Species** | **Island** | **Population** | **n** | **H_O_** | **uH_E_** | **F_IS_** |
| --- | --- | --- | --- | --- | --- | --- |
| Northern brushtail possum | Bathurst | BAL | 8 | 0.195 | 0.234 | 0.166 |
|  |  | Cape Fourcroy Grid | 15 | 0.189 | 0.231 | 0.182 |
|  |  | Cape Fourcroy West | 11 | 0.189 | 0.230 | 0.176 |
|  |  | East ROK | 7 | 0.194 | 0.231 | 0.161 |
|  |  | NG North | 5 | 0.191 | 0.228 | 0.163 |
|  |  | NG South | 3 | 0.191 | 0.221 | 0.133 |
|  |  | North Ranku | 7 | 0.194 | 0.233 | 0.167 |
|  |  | Ranku Barge | 3 | 0.191 | 0.225 | 0.153 |
|  |  | Ranku Grid | 18 | 0.195 | 0.237 | 0.177 |
|  |  | South Ranku | 4 | 0.195 | 0.230 | 0.153 |
|  |  | South ROK | 6 | 0.187 | 0.230 | 0.184 |
|  |  | West Ranku | 10 | 0.192 | 0.233 | 0.175 |
|  |  | West ROK | 8 | 0.192 | 0.229 | 0.162 |
|  | Melville | 3ways | 4 | 0.192 | 0.228 | 0.157 |
|  |  | Cache Point | 5 | 0.197 | 0.238 | 0.174 |
|  |  | College | 3 | 0.196 | 0.234 | 0.161 |
|  |  | Condor Point | 6 | 0.184 | 0.240 | 0.235 |
|  |  | East Melville | 6 | 0.184 | 0.234 | 0.212 |
|  |  | Goose | 54 | 0.194 | 0.239 | 0.185 |
|  |  | Jessie | 16 | 0.192 | 0.216 | 0.150 |
|  |  | Pickataramoor | 10 | 0.196 | 0.243 | 0.193 |
|  |  | Tony | 5 | 0.184 | 0.237 | 0.224 |
|  |  | West Johnson | 7 | 0.166 | 0.218 | 0.233 |
| Northern brown bandicoot | Bathurst | Cape Fourcroy West | 4 | 0.191 | 0.226 | 0.154 |
|  |  | Ranku Grid | 9 | 0.193 | 0.243 | 0.205 |
|  | Melville | Cache Point | 14 | 0.218 | 0.266 | 0.180 |
|  |  | College | 3 | 0.220 | 0.258 | 0.147 |
|  |  | Condor Point | 5 | 0.220 | 0.267 | 0.177 |
|  |  | Goose | 50 | 0.226 | 0.267 | 0.156 |
|  |  | Jessie | 27 | 0.218 | 0.256 | 0.149 |
|  |  | Pines Central | 5 | 0.220 | 0.263 | 0.162 |
|  |  | Snake Bay | 6 | 0.211 | 0.252 | 0.164 |
|  |  | Van Diemen | 3 | 0.214 | 0.249 | 0.142 |
|  |  | West Johnson | 5 | 0.202 | 0.254 | 0.206 |
| Black-footed tree-rat | Melville | Cache Point | 8 | 0.068 | 0.251 | 0.730 |
|  |  | Goose | 35 | 0.068 | 0.265 | 0.745 |
|  |  | Jessie | 39 | 0.068 | 0.263 | 0.743 |
|  |  | West Johnson | 6 | 0.072 | 0.248 | 0.712 |
|  |  | Pickataramoor | 13 | 0.071 | 0.263 | 0.732 |
|  |  | Pines | 4 | 0.071 | 0.233 | 0.697 |
|  |  | Snake Bay | 3 | 0.076 | 0.211 | 0.640 |

Table S3: Correlation matrix (Pearson’s correlation coefficients) between environmental layers tested.

|  | Vegetation | Fire frequency | Late fire frequency | Time since fire | Rainfall | Ruggedness | Distance to water | Distance to coast | Cat activity | Herbivore activity | Elevation |
| --- | --- | --- | --- | --- | --- | --- | --- | --- | --- | --- | --- |
| **Vegetation** | 1.00 |  |  |  |  |  |  |  |  |  |  |
| **Fire frequency** | -0.16 | 1.00 |  |  |  |  |  |  |  |  |  |
| **Late fire frequency** | -0.11 | 0.50 | 1.00 |  |  |  |  |  |  |  |  |
| **Time since fire** | 0.16 | -0.58 | -0.34 | 1.00 |  |  |  |  |  |  |  |
| **Rainfall** | -0.01 | -0.11 | -0.39 | 0.08 | 1.00 |  |  |  |  |  |  |
| **Ruggedness** | 0.04 | -0.14 | -0.14 | 0.11 | 0.05 | 1.00 |  |  |  |  |  |
| **Distance to water** | -0.05 | -0.13 | -0.13 | 0.13 | 0.06 | 0.00 | 1.00 |  |  |  |  |
| **Distance to coast** | -0.11 | 0.52 | 0.42 | -0.31 | -0.22 | -0.02 | -0.13 | 1.00 |  |  |  |
| **Cat activity** | 0.04 | 0.13 | 0.23 | -0.01 | -0.12 | -0.04 | -0.10 | 0.18 | 1.00 |  |  |
| **Herbivore activity** | -0.02 | 0.24 | 0.37 | -0.17 | -0.36 | -0.06 | -0.13 | 0.24 | 0.38 | 1.00 |  |
| **Elevation** | -0.21 | 0.48 | 0.24 | -0.30 | -0.05 | 0.13 | -0.01 | 0.73 | 0.08 | 0.17 | 1.00 |

Table S4: Pairwise population genomic differentiation (F_ST_) between all Bathurst Island populations of the northern brushtail possum with *n* ≥3.

|  | **N** | **Ranku Grid** | **Cape Fourcroy Grid** | **Cape Fourcroy West** | **NG North** | **NG South** | **North Ranku** | **South Ranku** | **BAL** | **Ranku Barge** | **West Ranku** | **South ROK** | **West ROK** | **East ROK** |
| --- | --- | --- | --- | --- | --- | --- | --- | --- | --- | --- | --- | --- | --- | --- |
| **Ranku Grid** | 18 | 0 |  |  |  |  |  |  |  |  |  |  |  |  |
| **Cape Fourcroy Grid** | 15 | 0.030 | 0 |  |  |  |  |  |  |  |  |  |  |  |
| **Cape Fourcroy West** | 11 | 0.029 | 0.011 | 0 |  |  |  |  |  |  |  |  |  |  |
| **NG North** | 5 | 0.015 | 0.032 | 0.031 | 0 |  |  |  |  |  |  |  |  |  |
| **NG South** | 3 | 0.018 | 0.037 | 0.036 | 0.003 | 0 |  |  |  |  |  |  |  |  |
| **North Ranku** | 7 | 0.009 | 0.036 | 0.036 | 0.024 | 0.027 | 0 |  |  |  |  |  |  |  |
| **South Ranku** | 4 | 0.000 | 0.028 | 0.028 | 0.011 | 0.015 | 0.005 | 0 |  |  |  |  |  |  |
| **BAL** | 8 | 0.016 | 0.029 | 0.030 | 0.007 | 0.012 | 0.020 | 0.014 | 0 |  |  |  |  |  |
| **Ranku Barge** | 3 | 0.004 | 0.035 | 0.034 | 0.017 | 0.021 | 0.006 | 0.002 | 0.022 | 0 |  |  |  |  |
| **West Ranku** | 10 | 0.007 | 0.036 | 0.034 | 0.025 | 0.027 | 0.007 | 0.005 | 0.021 | 0.001 | 0 |  |  |  |
| **South ROK** | 6 | 0.011 | 0.039 | 0.039 | 0.028 | 0.031 | 0.004 | 0.010 | 0.025 | 0.009 | 0.011 | 0 |  |  |
| **West ROK** | 8 | 0.018 | 0.047 | 0.048 | 0.036 | 0.041 | 0.017 | 0.021 | 0.033 | 0.015 | 0.017 | 0.009 | 0 |  |
| **East ROK** | 7 | 0.014 | 0.045 | 0.044 | 0.034 | 0.035 | 0.014 | 0.016 | 0.030 | 0.016 | 0.017 | 0.007 | 0.016 | 0 |

Table S5: Pairwise population genomic differentiation (F_ST_) between all Melville Island populations of the northern brushtail possum with *n* ≥3.

|  | **N** | **Condor Point** | **East Melville** | **Jessie** | **Goose** | **Johnson** | **Tony** | **Cache Point** | **Picka** | **3ways** | **College** |
| --- | --- | --- | --- | --- | --- | --- | --- | --- | --- | --- | --- |
| **Condor Point** | 6 | 0 |  |  |  |  |  |  |  |  |  |
| **East Melville** | 6 | 0.007 | 0 |  |  |  |  |  |  |  |  |
| **Jessie** | 16 | 0.022 | 0.030 | 0 |  |  |  |  |  |  |  |
| **Goose** | 54 | 0.014 | 0.020 | 0.011 | 0 |  |  |  |  |  |  |
| **Johnson** | 7 | 0.020 | 0.028 | 0.036 | 0.028 | 0 |  |  |  |  |  |
| **Tony** | 5 | 0.005 | 0.013 | 0.022 | 0.011 | 0.020 | 0 |  |  |  |  |
| **Cache Point** | 5 | 0.022 | 0.032 | 0.007 | 0.010 | 0.037 | 0.021 | 0 |  |  |  |
| **Picka** | 10 | 0.018 | 0.026 | 0.024 | 0.015 | 0.034 | 0.014 | 0.027 | 0 |  |  |
| **3ways** | 4 | 0.036 | 0.044 | 0.041 | 0.031 | 0.049 | 0.035 | 0.046 | 0.030 | 0 |  |
| **College** | 3 | 0.009 | 0.020 | 0.017 | 0.007 | 0.020 | 0.004 | 0.019 | 0.006 | 0.031 | 0 |

Table S6: Pairwise population genomic differentiation (F_ST_) between all Bathurst Island populations of the northern brown bandicoot with *n* ≥3.

|  | **N** | **Ranku Grid** | **Cape Fourcroy West** |
| --- | --- | --- | --- |
| **Ranku Grid** | 9 | 0 |  |
| **Cape Fourcroy West** | 4 | 0.038 | 0 |

Table S7: Pairwise population genomic differentiation (F_ST_) between all Melville Island populations of the northern brown bandicoot with *n* ≥3.

|  | **N** | **Van Diemen** | **Condor** | **Jessie** | **Goose** | **Johnson** | **Pines** | **Cache Point** | **Snake Bay** | **College** |
| --- | --- | --- | --- | --- | --- | --- | --- | --- | --- | --- |
| **Van Diemen** | 7 | 0 |  |  |  |  |  |  |  |  |
| **Condor** | 5 | 0.055 | 0 |  |  |  |  |  |  |  |
| **Jessie** | 27 | 0.052 | 0.024 | 0 |  |  |  |  |  |  |
| **Goose** | 50 | 0.048 | 0.017 | 0.008 | 0 |  |  |  |  |  |
| **Johnson** | 5 | 0.055 | 0.015 | 0.026 | 0.021 | 0 |  |  |  |  |
| **Pines** | 10 | 0.022 | 0.030 | 0.032 | 0.027 | 0.034 | 0 |  |  |  |
| **Cache Point** | 14 | 0.056 | 0.023 | 0.006 | 0.009 | 0.027 | 0.034 | 0 |  |  |
| **Snake Bay** | 6 | 0.029 | 0.054 | 0.051 | 0.046 | 0.054 | 0.019 | 0.055 | 0 |  |
| **College** | 3 | 0.056 | 0.024 | 0.024 | 0.017 | 0.031 | 0.022 | 0.024 | 0.052 | 0 |

Table S8: Pairwise population genomic differentiation (F_ST_) between all populations of the black-footed tree-rat with *n* ≥3.

|  | **N** | **Picka** | **Cache Point** | **Goose** | **Jessie** | **Snake Bay** | **Pines** | **Johnson** |
| --- | --- | --- | --- | --- | --- | --- | --- | --- |
| **Picka** | 13 | 0 |  |  |  |  |  |  |
| **Cache Point** | 8 | 0.021 | 0 |  |  |  |  |  |
| **Goose** | 35 | 0.014 | 0.009 | 0 |  |  |  |  |
| **Jessie** | 39 | 0.022 | 0.007 | 0.007 | 0 |  |  |  |
| **Snake Bay** | 3 | 0.020 | 0.041 | 0.028 | 0.036 | 0 |  |  |
| **Pines** | 4 | 0.012 | 0.035 | 0.023 | 0.033 | 0.002 | 0 |  |
| **Johnson** | 6 | 0.021 | 0.036 | 0.026 | 0.032 | 0.047 | 0.029 | 0 |

**Figures**


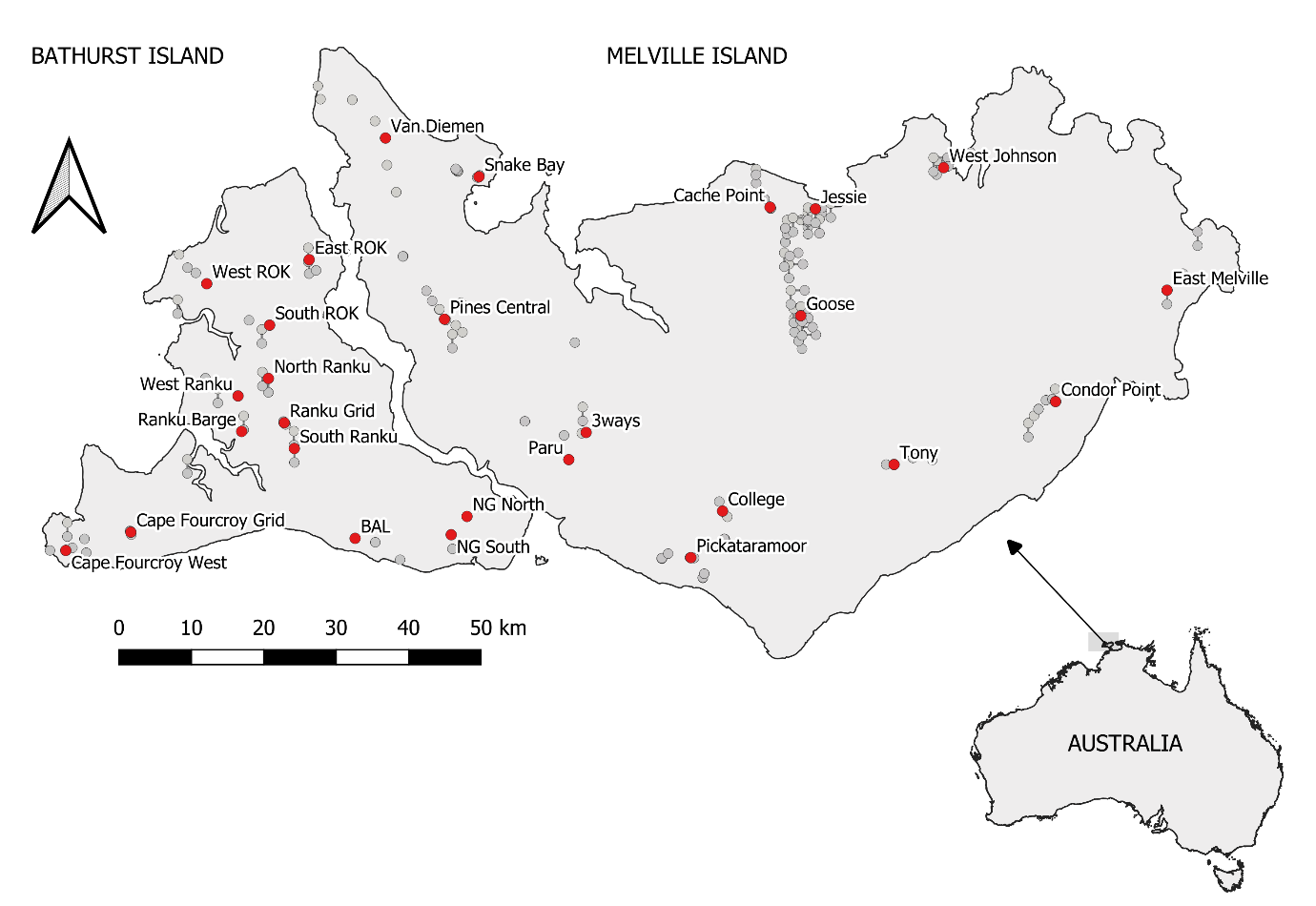


Figure S1: The locations of sampling sites (red points) relative to samples collected (grey points) for all species. Genetic diversity and structure values are only presented for sites with n ≥ 3. Landscape genetic analysis is individual based.

Figure S2a-k: Layers


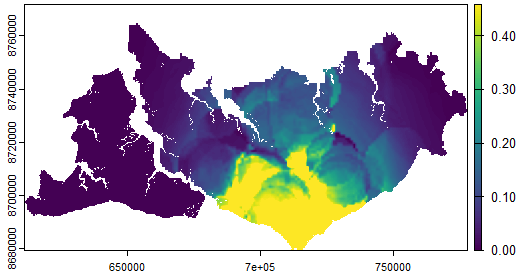


Figure S2a. Cat activity: Generalised Linear Model (GLM) of cat detections from camera trapping data producing a layer of predicted nightly trap success


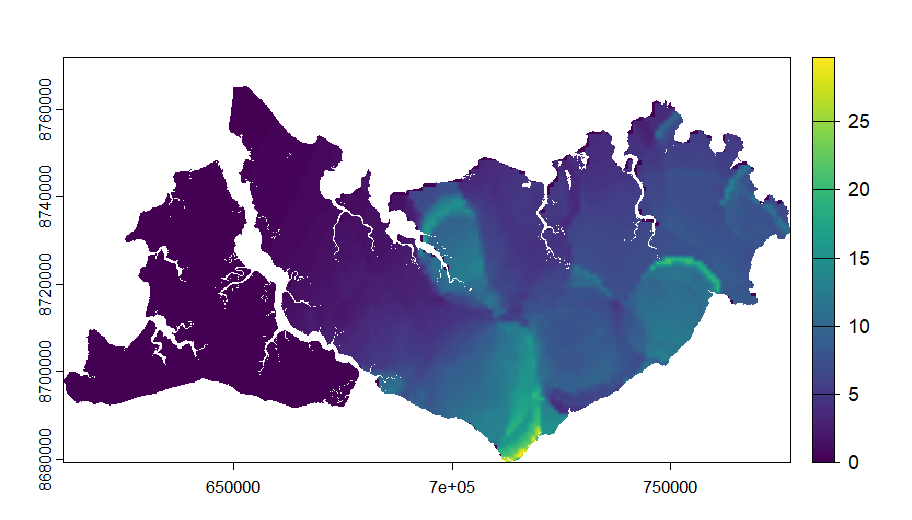


Figure S2b. Feral herbivore activity: Generalised Linear Model (GLM) of buffalo and horse detections from camera trapping data producing a layer of predicted nightly trap success


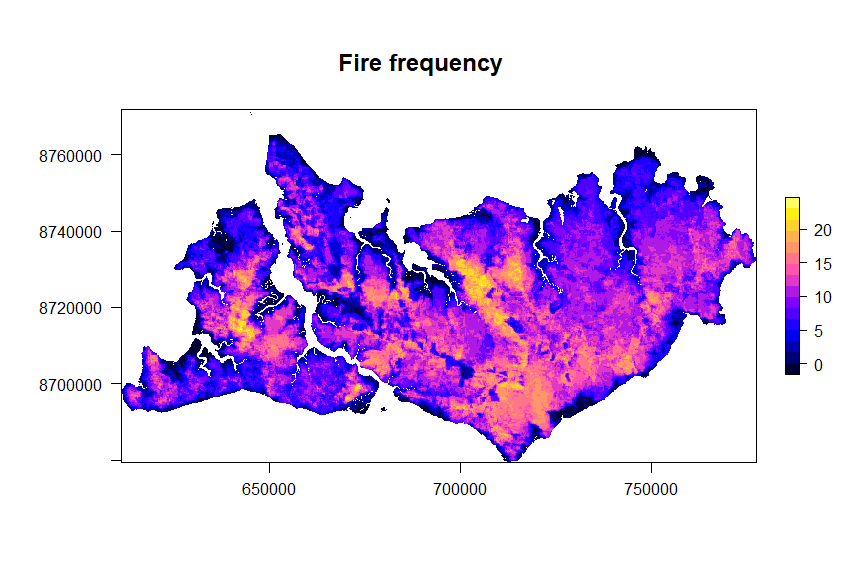


Figure S2c. Fire frequency: Number of years burnt between 2000-2023. Source: (NAFI, 2024)


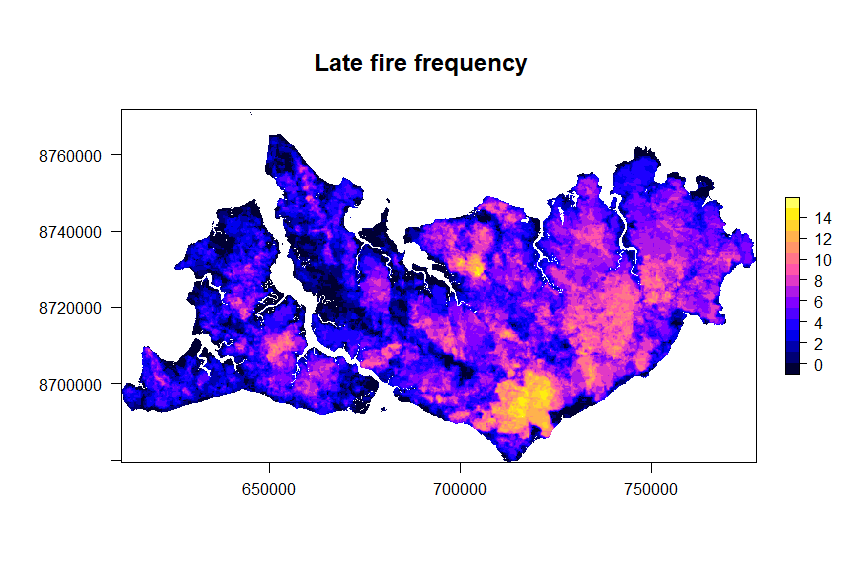


Figure S2d. Late fire frequency: Number of years burnt after July 31st between 2000-2023. Source: (NAFI, 2024)


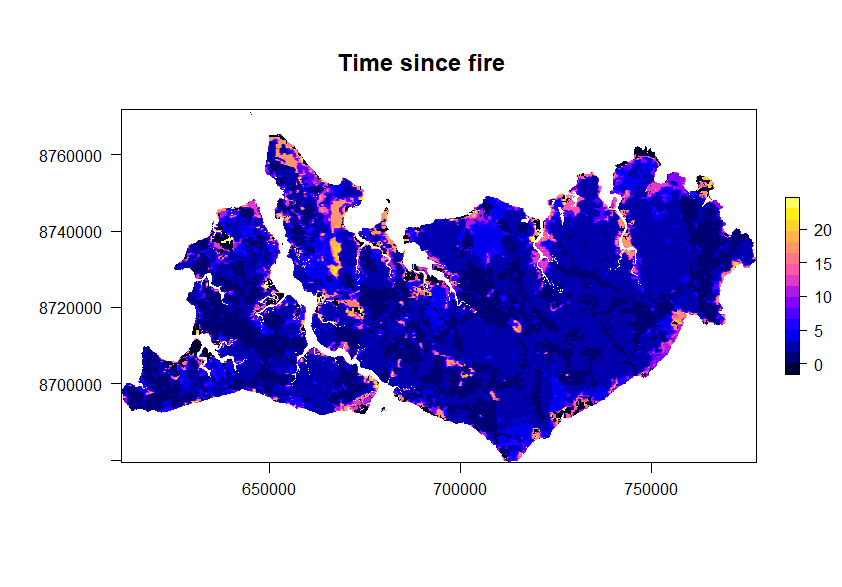


Figure S2e. Time since fire: Number of years since last burnt. Source: (NAFI, 2024)


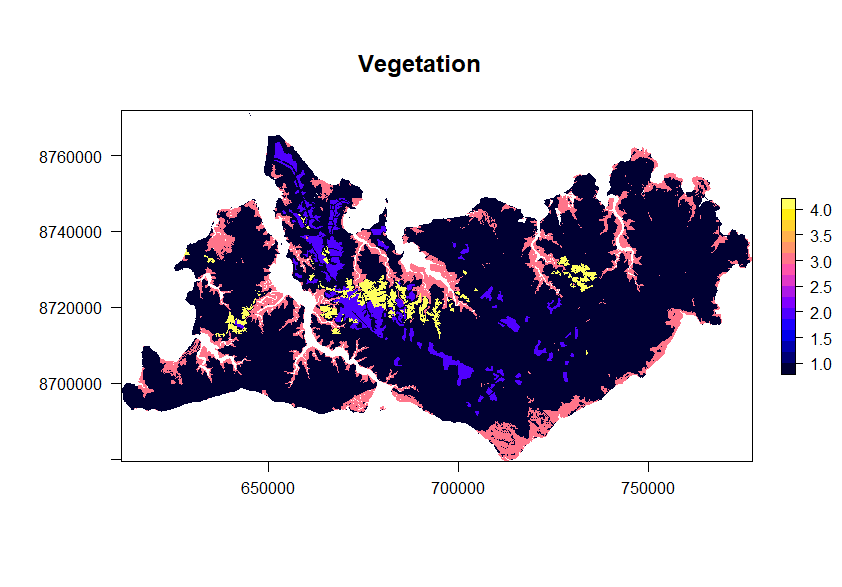


Figure S2f. Vegetation: Type of vegetation class. Displayed here is the simplified layer of four classes including 1) forest/woodland, 2) plantation, 3) treeless plains, and 4) salt areas.


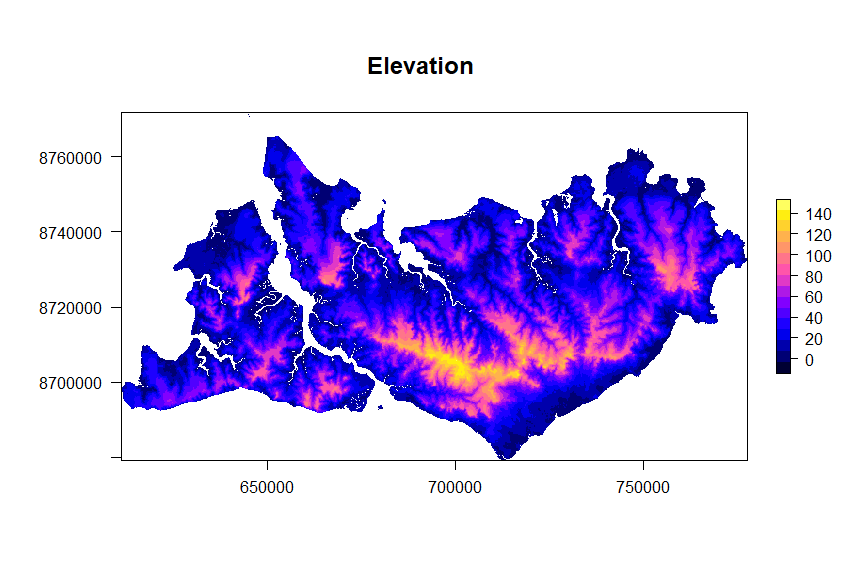


Figure S2g. Elevation: Derived from shuttle radar topography mission (SRTM) digital elevation model (DEM) with a 30-m resolution Source: (Geoscience Australia, 2024b)


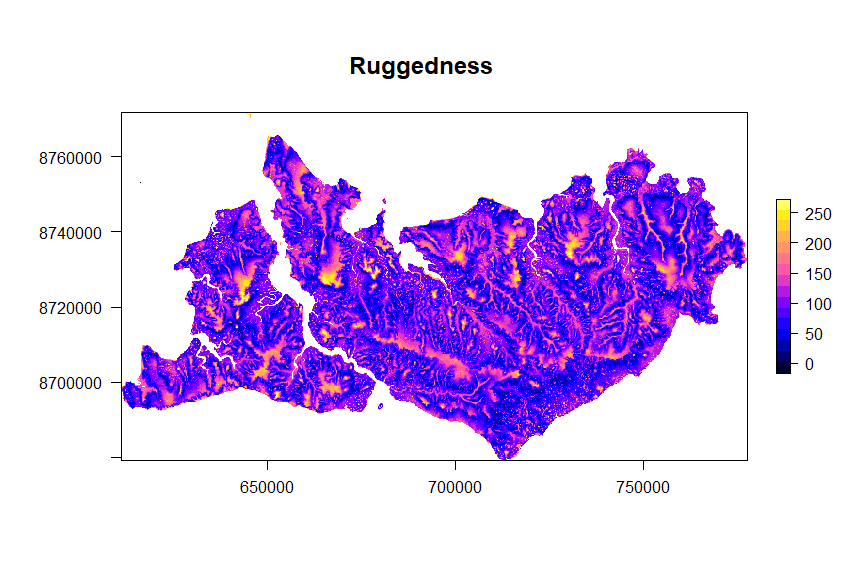


Figure S2h. Topographic ruggedness: Calculated from the difference in elevation between a cell and the eight cells surrounding it. Source: (Geoscience Australia, 2024a)


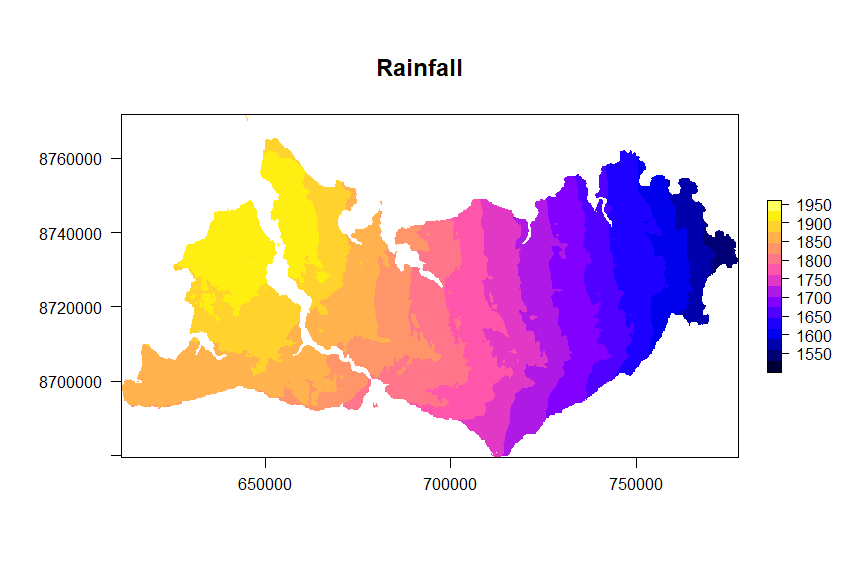


Figure S2i. Rainfall: Mean annual rainfall (mm). Source: (BIOCLIM, 2024)


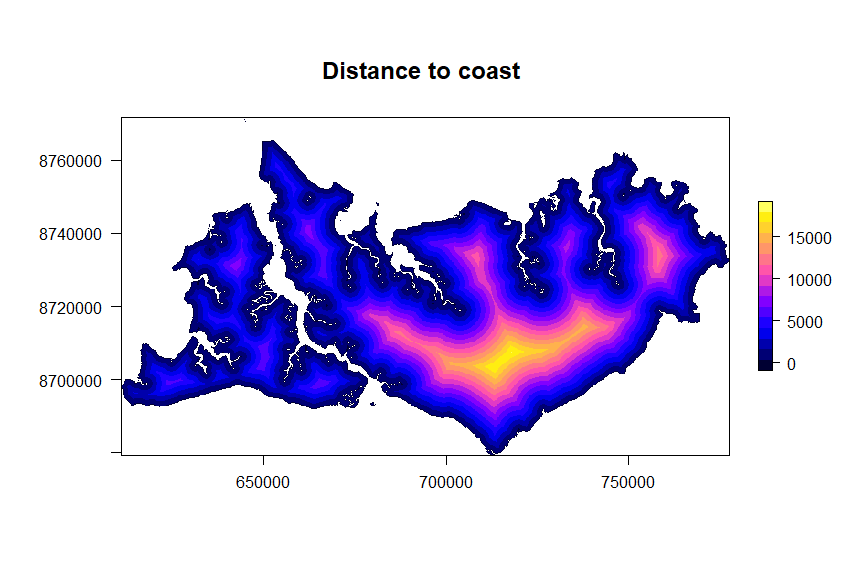


Figure S2j. Distance (m) to coast.


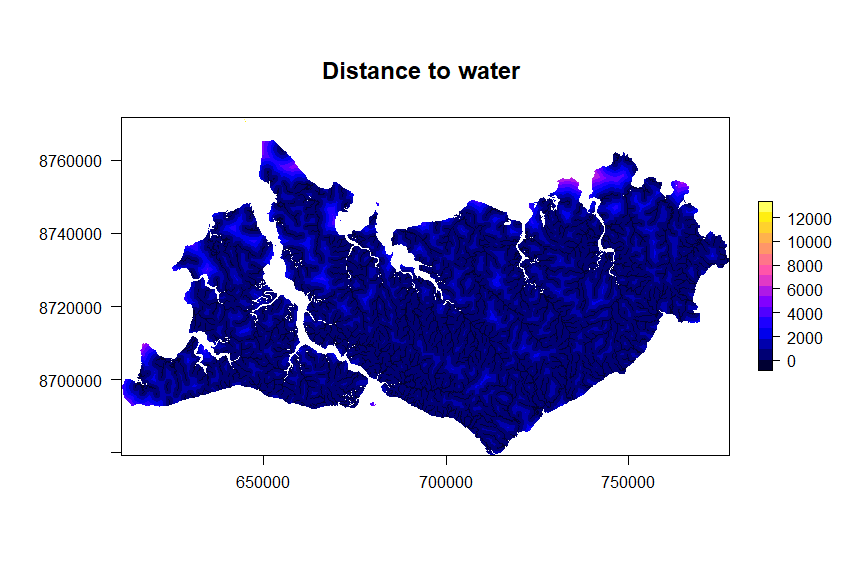


Figure S2k. Distance to water: Distance (m) to a perennial or non-perennial watercourse.


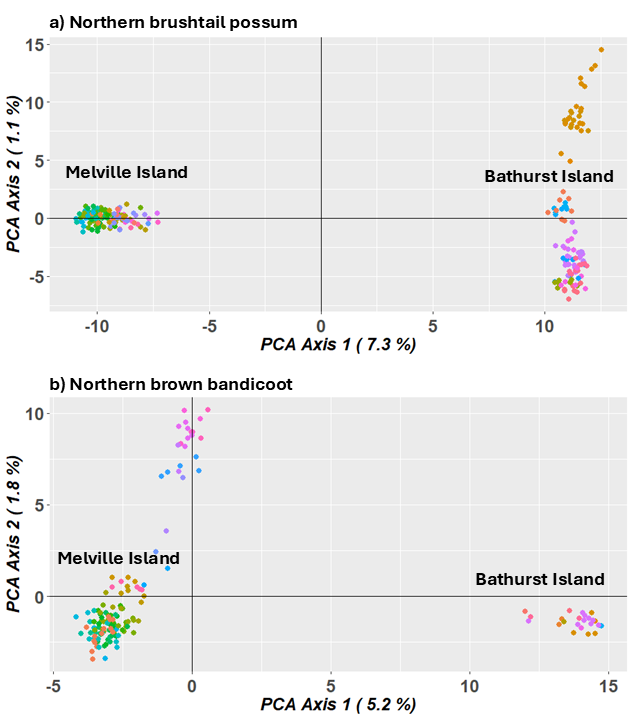


Figure S3: Comparison of principle coordinate axis (PCoA) 1 and 2, visualising genetic differences among individuals of (a) northern brushtail possums and (b) northern brown bandicoots across Bathurst and Melville Islands.


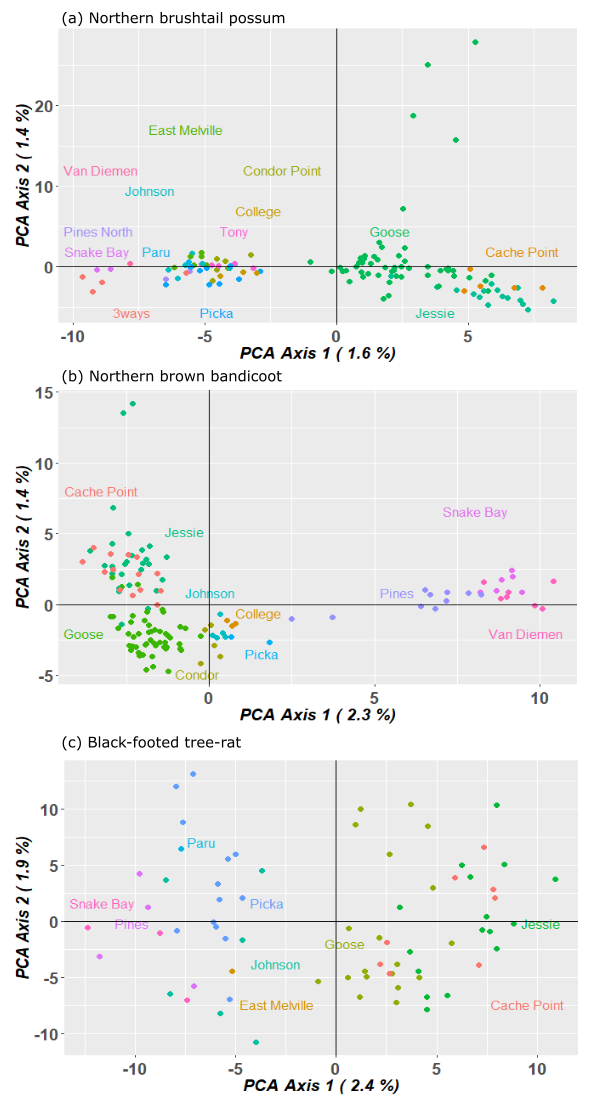


Figure S4: Comparison of principle coordinate axis (PCoA) 1 and 2, visualising genetic differences among individuals of (a) northern brushtail possums, (b) northern brown bandicoots, and (c) black-footed tree-rats on Melville Island.


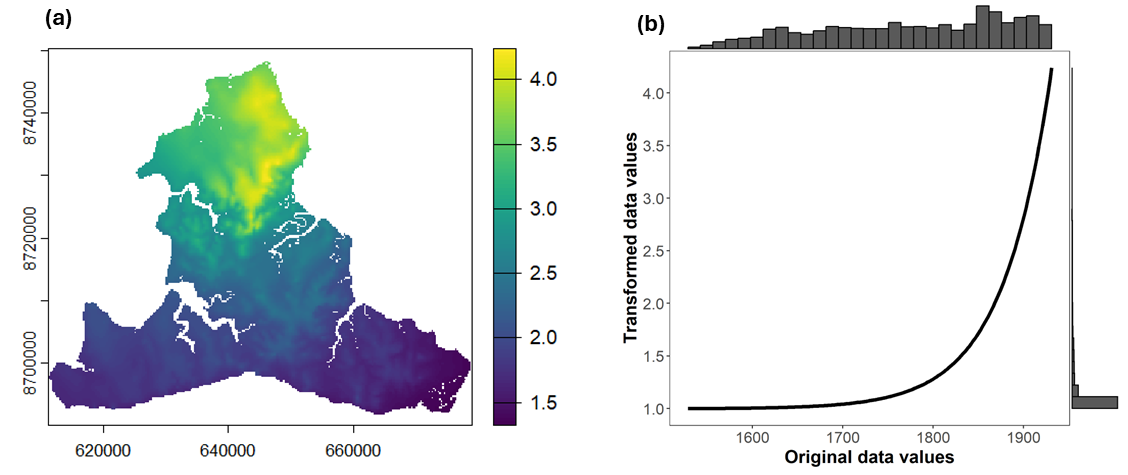


Figure S5: The top-ranked optimised resistance surface best explaining northern brushtail possum genetic structure on Bathurst Island at a fine-scale (< 20 km) was (a) rainfall, with the high rainfall in the north conferring more resistance to genetic connectivity as shown in the transformed data values (b).


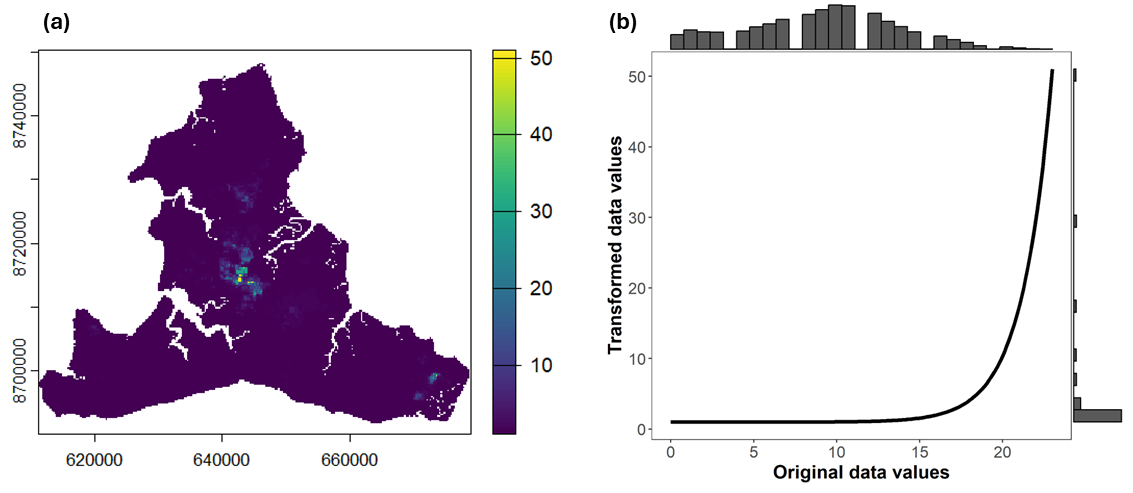


Figure S6: The top-ranked optimised resistance surface best explaining northern brown bandicoot genetic structure on Bathurst Island at a fine-scale (< 20 km) was (a) fire frequency, with the high fire frequency in the centre conferring more resistance to genetic connectivity and shown in the transformed data values (b).


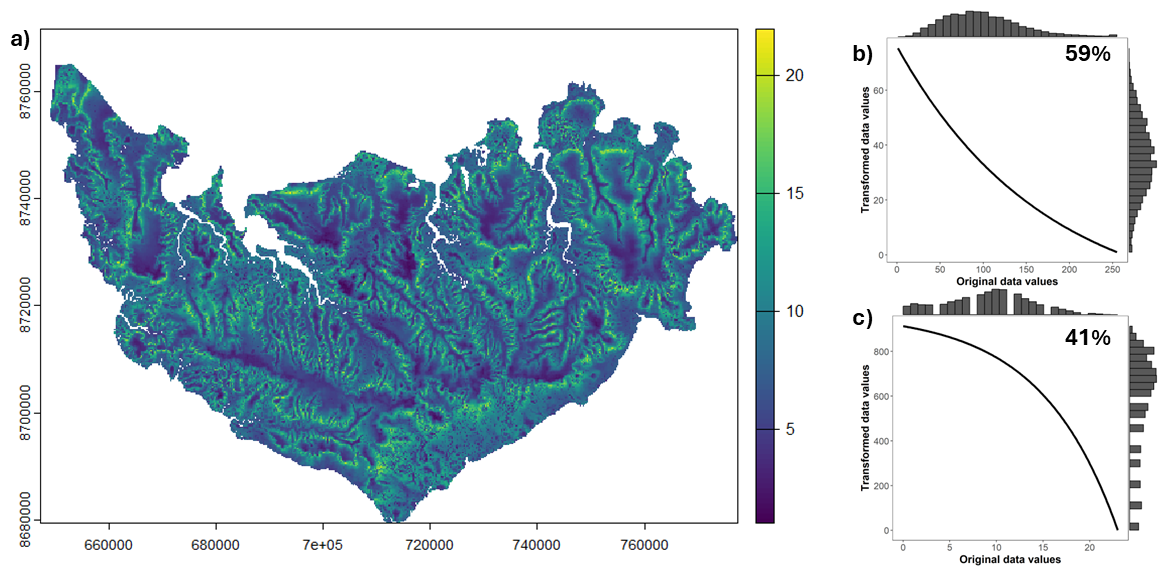


Figure S7: The top-ranked optimised resistance surface explaining northern brushtail possum genetic structure on Melville Island at a broad-scale was (a) the composite of topographic ruggedness and fire frequency. Both low topographic ruggedness (b) and low fire frequency (c) were associated with higher resistance to gene flow.


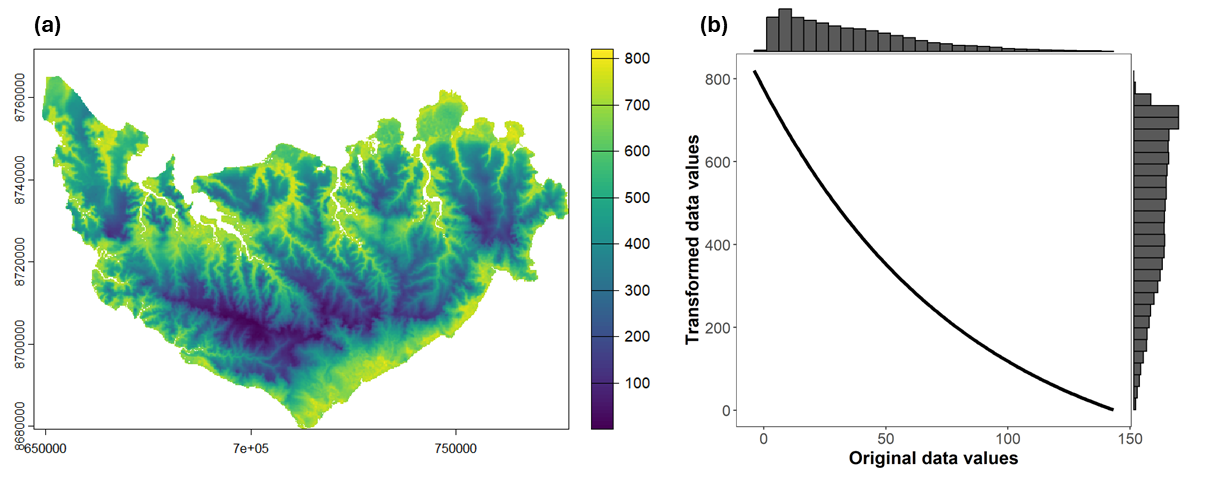


Figure S8: The second top-ranked optimised resistance surface (after geographic distance) best explaining northern brushtail possum genetic structure on Melville Island at a fine-scale (< 20 km) was (a) elevation, with areas of low elevation conferring more resistance to genetic connectivity and shown in the transformed data values (b).


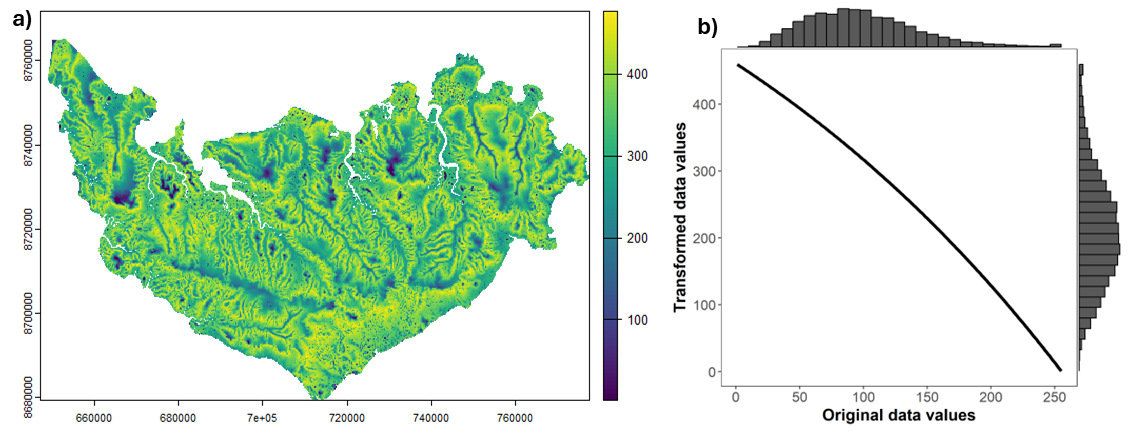


Figure S9: The top-ranked optimised resistance surface best explaining northern brown bandicoot genetic structure on Melville Island at a broad-scale was (a) topographic ruggedness, with low topographic ruggedness associated with high resistance to gene flow as shown in the transformed data values (b).


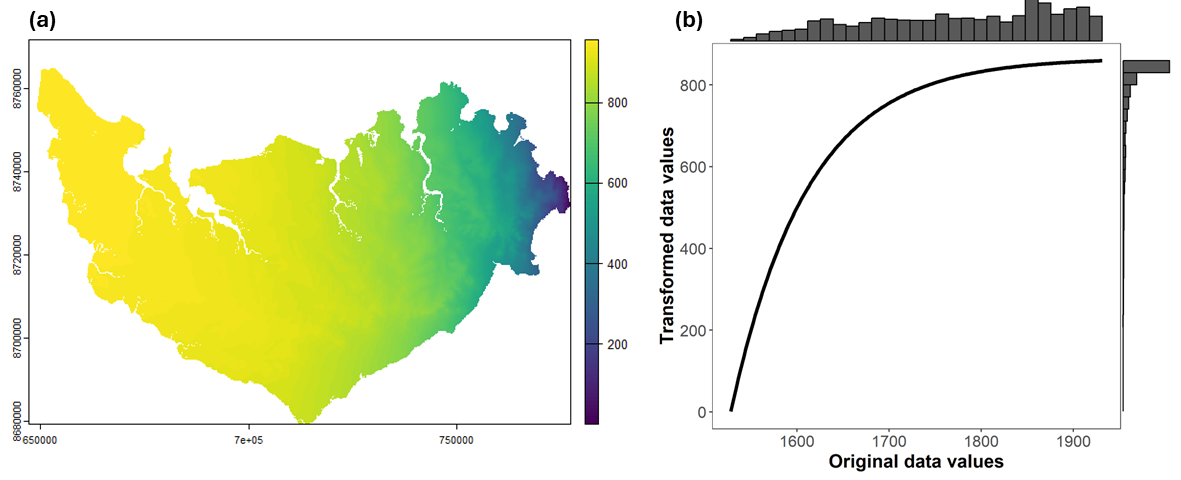


Figure S10: The second top-ranked optimised resistance surface (after geographic distance) best explaining northern brown bandicoot genetic structure on Melville Island at a fine-scale (< 20 km) was (a) rainfall, with the high rainfall in the west conferring more resistance to genetic connectivity and shown in the transformed data values (b).


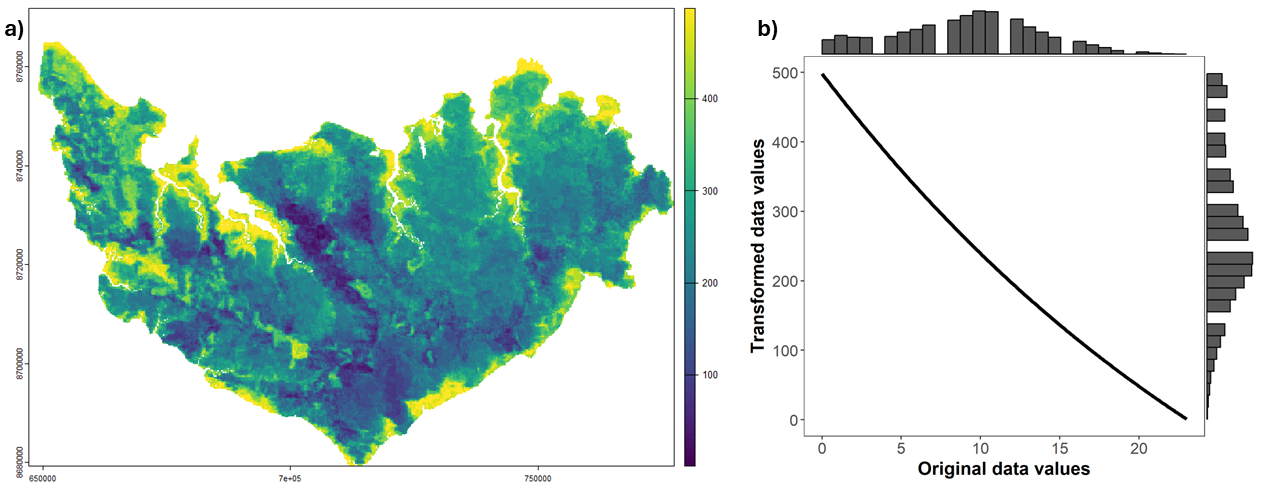


Figure S11: The top-ranked optimised single resistance surface best explaining black-footed tree-rat genetic structure on Melville Island at a broad-scale was (a) fire frequency, with the low fire frequency areas such as mangroves and plantations conferring more resistance to genetic connectivity and shown in the transformed data values (b).


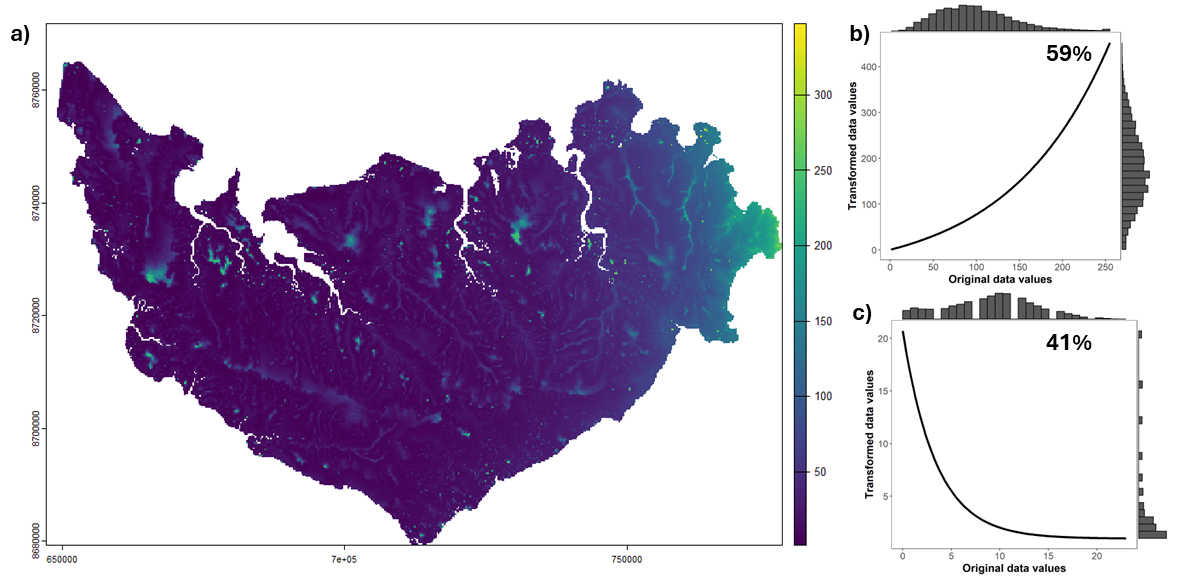


Figure S12: The top-ranked optimised single resistance surface best explaining black-footed tree-rat genetic structure on Melville Island at a fine-scale (< 20 km) was (a) the composite surface of topographic ruggedness and rainfall. High topographic ruggedness (b) and low rainfall (c) were associated with higher resistance to gene flow.

**References**

BIOCLIM. (2024). *Bioclimatic variables*. WorldClim. <https://www.worldclim.org/data/bioclim.html>

Geoscience Australia. (2024a). *Multi-scale Topographic Position Image of Australia*. Geoscience Australia. <https://ecat.ga.gov.au/geonetwork/srv/api/records/34dcb0bf-0fb4-42c2-bf9e-cbbd5449a1da>

Geoscience Australia. (2024b). *SRTM-derived 1 Second Digital Elevation Models Version 1.0*. Geoscience Australia. <https://ecat.ga.gov.au/geonetwork/srv/eng/catalog.search#/metadata/72759>

NAFI. (2024). *North Australia & Rangelands Fire Information* Charles Darwin University, Darwin. <https://firenorth.org.au/nafi3/>
